# A General Introduction to Glucocorticoid Biology

**DOI:** 10.3389/fimmu.2019.01545

**Published:** 2019-07-04

**Authors:** Steven Timmermans, Jolien Souffriau, Claude Libert

**Affiliations:** ^1^Center for Inflammation Research, VIB, Ghent, Belgium; ^2^Department of Biomedical Molecular Biology, Ghent University, Ghent, Belgium

**Keywords:** glucocorticoids, glucocorticoid receptor, inflammation, molecular biology, SEDIGRAM

## Abstract

Glucocorticoids (GCs) are steroid hormones widely used for the treatment of inflammation, autoimmune diseases, and cancer. To exert their broad physiological and therapeutic effects, GCs bind to the GC receptor (GR) which belongs to the nuclear receptor superfamily of transcription factors. Despite their success, GCs are hindered by the occurrence of side effects and glucocorticoid resistance (GCR). Increased knowledge on GC and GR biology together with a better understanding of the molecular mechanisms underlying the GC side effects and GCR are necessary for improved GC therapy development. We here provide a general overview on the current insights in GC biology with a focus on GC synthesis, regulation and physiology, role in inflammation inhibition, and on GR function and plasticity. Furthermore, novel and selective therapeutic strategies are proposed based on recently recognized distinct molecular mechanisms of the GR. We will explain the SEDIGRAM concept, which was launched based on our research results.

## Discovery of Glucocorticoids and the Glucocorticoid Receptor

The first steps leading to the discovery of glucocorticoids (GCs) took place in the 19th century when the physician Thomas Addison described that patients suffering from (chronic) fatigue, muscular degeneration, weight loss, and a strange darkening of the skin could obtain beneficial effects from adrenal extracts (1). This disease is now known as Addison's disease, which is a form of adrenal insufficiency. In 1946, Edward Calvin Kendall isolated four steroidal compounds from adrenal extracts, which he named compounds A, B, E, and F (2). Compound E, would become known as cortisol and was synthesized later that year by Sarett (3). The therapeutic potential was discovered by rheumatologist Philip Hench in a patient suffering from rheumatoid arthritis (4). Hench and Kendall were awarded the Nobel prize for Medicine and Physiology in 1950 together with Tadeus Reichstein who succeeded in isolating several steroid hormones from the adrenals, eventually leading to the discovery of cortisol. Since the discovery of their anti-inflammatory potential GCs were hailed as wonder drugs to treat various inflammatory diseases and became part of the group of most used and cost-effective anti-inflammatory drugs.

GCs bind the GC receptor (GR), a member of the nuclear receptor (NR) family of intracellular receptors, which also contains the estrogen receptor (ER), progesterone receptor (PR), androgen receptor (AR), and mineralocorticoid receptor (MR) as well as several orphan receptors (with no known ligand) (5, 6). In 1966, the GR was identified as the principal receptor responsible for the physiological and pharmacological effects of GCs (7). It would take almost two more decades for the human GR-coding gene, *NR3C1* to be cloned (8, 9). The GR is very closely related to the MR and these receptors exhibit some cross-reactivity, more specifically the MR is activated both by its own ligands, mineralocorticoids (MCs) and by GCs, but GR is activated only by GCs (10). NRs are involved in many aspects of mammalian biology, including various metabolic functions, cardiac function, reproduction and (embryonic) development, and the immune system (11).

## Glucocorticoid Synthesis, Regulation and Biological Availability

GCs are steroid hormones that are essential for the daily functioning of mammals. They are involved in several physiological processes, namely in metabolism (12), water and electrolyte balance (13), the immune response (14, 15), growth (16), cardiovascular function (17, 18), mood and cognitive functions (19–21), reproduction (22), and development (23). GCs are mainly synthesized in the cortex of the adrenal gland together with aldosterone (a MC) and dehydro-epi-androsterone (DHEA). The latter is the precursor of testosterone and estrogen. Aldosterone, GCs, and DHEA are synthesized by different steroidogenic enzymes in the mitochondria of, respectively, the zona glomerulosa, the zona fasciculate, and the zona reticularis of the adrenal cortex. They are however all synthesized from the same precursor, namely cholesterol (24). Extra-adrenal GC production in the thymus, vasculature, brain, and epithelial barriers has also been observed (25–30). These locally produced GCs are thought to predominantly exert local effects and contribute only minimally to the systemically circulating pool of GCs allowing a high spatial specificity of steroid actions, which are also independent of the circadian and stress induced regulation of endogenous GCs.

**Figure 1:**
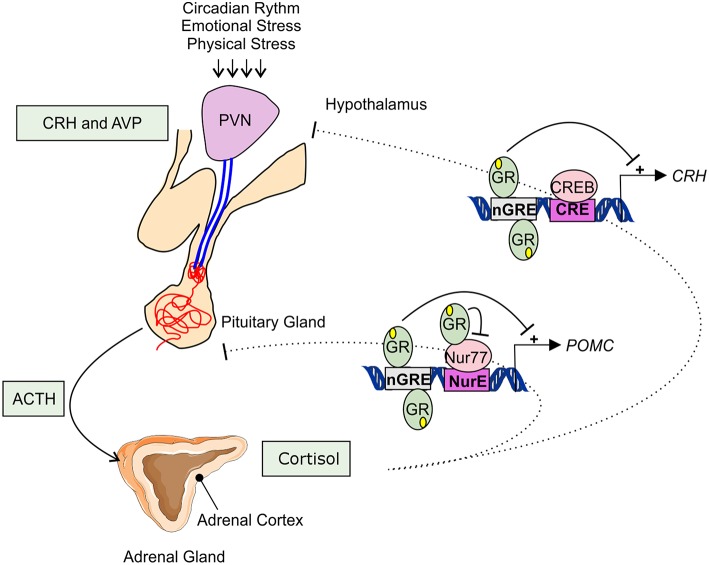
Hypothalamic-pituitary-adrenal axis. The hypothalamic-pituitary-adrenal (HPA) axis activity is controlled by the circadian rhythm and can be induced by physiological and emotional stress. When activated, corticotrophin-releasing hormone (CRH), and arginine vasopressin (AVP) are released from the hypothalamic paraventricular nucleus (PVN). This induces the release of adrenocorticotrophic hormone (ACTH) from the pituitary gland into the systemic circulation. ACTH will activate cortisol synthesis in the cortex of the adrenal gland. Cortisol negatively regulates the HPA-axis activity, e.g., by repressing the transcription of *CRH* and *POMC* by binding to negative glucocorticoid responsive elements (nGRE) or by binding to the transcription factor Nur77 involved in the *POMC* expression.

Adrenal GC production is regulated by the hypothalamic-pituitary-adrenal (HPA) axis (Figure 1). Under basal, unstressed conditions GCs are released from the adrenal glands in the bloodstream in a circadian and ultradian rhythm characterized by peak levels during the active phase which is in the morning in humans and in the beginning of nighttime in nocturnal animals such as mice. The activity of the HPA axis is further increased upon physiological (e.g., activated immune response) and emotional stress. When the HPA-axis is stimulated, corticotropin-releasing hormone (CRH), and arginine vasopressin (AVP) are released from the hypothalamic paraventricular nucleus (PVN). Subsequently, CRH and AVP bind their receptor CRH-R1 and V1B in the anterior pituitary inducing the release of adrenocorticotrophic hormone (ACTH) in the circulation. ACTH will in turn stimulate the adrenal gland to synthetize and secrete GC hormones (cortisol) in the circulation (31).

The HPA axis is subject to a negative feedback inhibition by GCs, both in a genomic and a non-genomic way. The genomic feedback regulation is mediated through binding of GCs to the GR both at the level of the PVN and the pituitary gland, thereby repressing the *CRH, CRH-R1*, and the *POMC* gene (Figure 1). *POMC* codes for the proopiomelanocortin prohormone which is the precursor of ACTH. *CRH, CRH-R1*, and *POMC* gene expression are repressed by the binding of GR to negative glucocorticoid responsive elements (nGREs) (32–34). Next to this, GR is also able to physically interact with the Nur77 protein which also binds in the POMC promoter, thereby preventing it from performing its transcription function (35, 36). Non-genomically, GCs regulate the HPA axis for example via the release of endocannabinoid from CRH neurons thereby suppressing the release of glutamate from presynaptic excitatory synapses (37), or via γ-aminobutyric acid (GABA) release at the inhibitory synapses of CRH neurons (38).

Once secreted in the bloodstream GCs are bound to and transported by plasma proteins which keep the GCs inactive. Corticosteroid-binding globulin (CBG) is the main GC-binding protein in the plasma, with about 80–90% of the GCs bound to it (39). Several proteases target CBG, such as neutrophil elastase at sites of infection (40), causing the release of bound GCs. Approximately 10% of the GCs are bound to albumin that binds GCs with less affinity than CBG (39).

**Figure 2:**
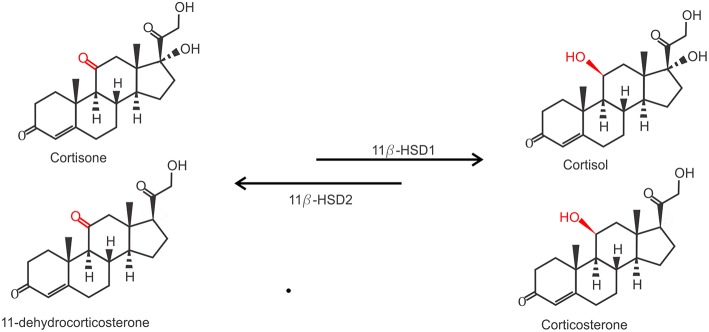
Conversion of inactive GCs to active GCs. Inactive Cortisone (human) and 11-dehydrocorticosterone (mouse) are activated to active cortisol and corticosterone by 11β-hydroxysteroid dehydrogenase 1 (11β-HSD1), and inactivated again by 11β-HSD2.

Due to their lipophilic nature, free GCs diffuse through the cell membrane to exert their function. However, the actual bioavailability of GCs in the cytoplasm is regulated by the balance between active and inactive forms of GCs. Two enzymes are responsible for the conversion between inactive cortisone (or 11-dehydrocorticosterone in mice) on the one hand and the active cortisol (or corticosterone, in mice) on the other hand. While 11β-hydroxysteroid dehydrogenase 1 (11β-HSD1) catalyzes the conversion of cortisone to cortisol, 11β-HSD2 carries out the opposite reaction (Figure 2). 11β-HSD2 is highly expressed in tissues with high MR expression, such as the kidneys, to prevent GC-induced MR activation which is known to cause salt and water dyshomeostasis (41, 42). Biologically active GCs will bind their receptor in the cytoplasm which exerts their physiological effects. This mechanism also confers a tight spatial regulation of GC actions, as the levels of these enzymes may be tissue or even cell specifically regulated and will directly determine the balance between the inactive and active form of GCs and thus the strength of the effect.

Under physiological conditions the role of endogenous GCs is not simply anti-inflammatory or immunosuppressive and shows more immunomodulation. It has been shown that GCs can also work pro-inflammatory (14). This occurs mainly in conditions of acute stress and is related to the concentration of GCs present (14, 43). Such pro-inflammatory actions were shown to include: elevation of pro-inflammatory cytokine levels (IL-1β) (44) or an exacerbation of the peripheral immune response in delayed type hypersensitivity (45).

Next to the endogenous GCs, various synthetic GCs (e.g., Prednisolone, Methylprednisolone, Fluticasone, Budesonide, and Dexamethasone) have been developed by the pharmaceutical industry that serve as treatments for various diseases. All these synthetic GCs were developed based on the structure of endogenous GCs (cortisol/hydrocortisone) (46). Experiments with structural modifications, mainly replacing side chains, resulted in synthetic GCs with optimized characteristics for medical use (pharmacokinetics, bioavailability, cross-reactivity with the MR). The most obvious differences between synthetic and endogenous GCs are (i) potency, as the synthetic variants are usually much better activators of the receptor than cortisol (4x−80x more) (47). (ii) Specificity, since endogenous GCs activate both GR and MR, but many synthetic GCs (e.g., dexamethasone, methylprednisolone) act (almost) exclusively on the GR. And (iii) synthetic GCs may (prednisolone) or may not (dexamethasone) be subject to processing by 11β-HSD1/2 which has a major impact on their bioavailability, as some synthetic GCs may (not) need to be activated by these enzymes or cannot be changed into an inactive form by them. Also, most synthetic GCs also do not bind the carrier proteins such as CBG (48–50). These facts are important to keep in mind when giving GC treatment or performing research using synthetic GCs.

## The Glucocorticoid Receptor

The GR mediates the actions of GCs in cells. It belongs to the nuclear receptor superfamily of transcription factors (TFs) and is a 97 kDa protein that is constitutively and ubiquitously expressed throughout the body (51). Nevertheless, GCs exert cellular and tissue-specific effects due to the existence of different GR isoforms on the one hand and cell- and context-specific allosteric signals influencing GR function on the other hand (52–54). The GR functions by regulating the expression of GC responsive genes in a positive or negative manner. It is estimated that there are between 1,000 and 2,000 genes that are subject to GR mediated regulation, with some studies stating that up to 20% of all genes are responsive to the GR in some way (55).

### GR Gene and Protein

The human gene encoding the GR is the “nuclear receptor subfamily 3 group c member 1” (*NR3C1*) gene localized on chromosome 5 (5q31.3). The mouse *Nr3c1* gene is localized on chromosome 18. The hGR gene consists of 9 exons of which exon 1 forms the 5′ untranslated region (UTR) and exons 2–9 encode the GR protein (52).

**Figure 3:**
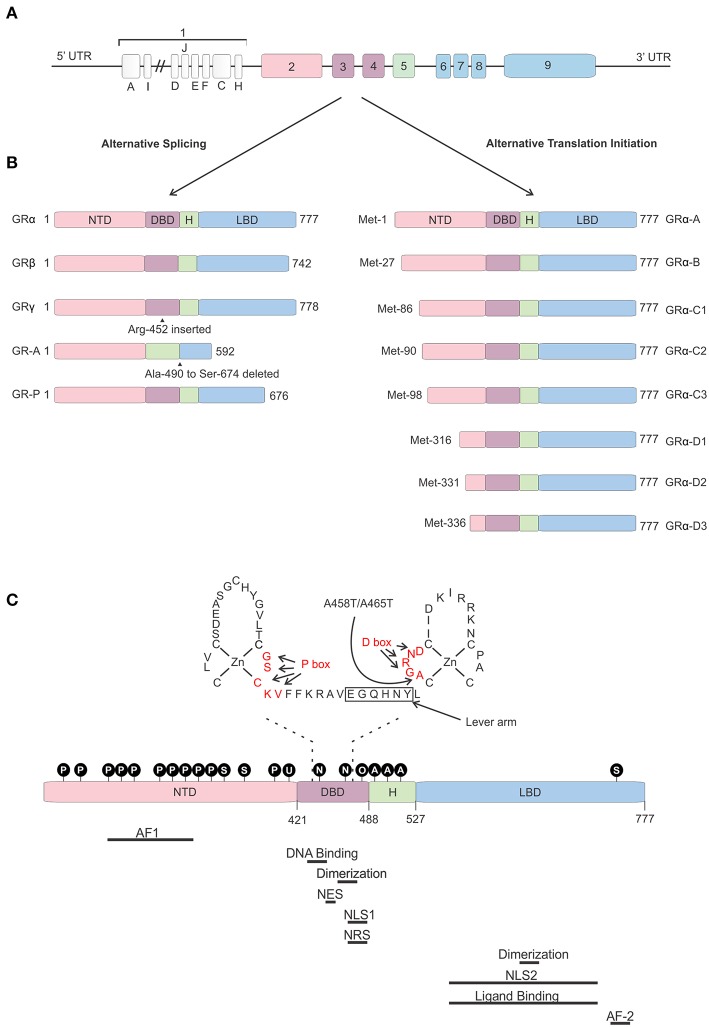
Glucocorticoid receptor gene and protein. **(A)** Genomic structure of the glucocorticoid receptor (GR) gene. **(B)** Alternative splice and translation-initiation variants of the GR protein. **(C)** Structure of the GR protein consisting of an N-terminal domain (NTD), DNA-binding domain (DBD), a hinge region (H), and a ligand-binding domain (LBD), with a focus on the two zinc-fingers of the DBD and the GR^Dim^ mutation (A458T in human, A465T in mouse). Identified post-translational modifications of the GR are indicated in the black circles. Regions important in GR function are indicated below the protein. AF, Activation function; NES, Nuclear Export Signal; NLS, Nuclear Localization Signal; NRS, Nuclear Retention Signal; P, phosphorylation; S, sumoylation; U, ubiquitination; N, nitrosylation; O, oxidation; A, acetylation.

The 5′ UTR of the hGR is GC-rich, but does not contain TATA or CAT boxes (56). Thus, far 13 hGR exon 1 variants differing in upstream promoter regions have been identified (A1–3, B, C1–3, D–F, H–J) (Figure 3). Differential use of these promoters, located about 5 kb upstream of the transcription start site, causes varying expression levels of GR protein isoforms between cells and tissues (57–60). These promoters contain multiple binding sites for several TFs such as AP-1 (61) and Interferon Regulatory Factor (IRF) (62), but also for GR itself, thereby enabling the regulation of its own expression (63). Furthermore, these exon-1 variants are subject to epigenetic regulation. Several epigenetic modifications, such as DNA methylation and histone acetylation/methylation are known to occur in this region (or in other regions). The presence or absence of such modifications has been related to GR gene expression levels, GC resistance in certain cancers, promotion of cancer development, and mental health (64–69).

The hGR protein (Figure 3) is a modular protein that, like other NR family proteins, is built up out of an amino-terminal domain (NTD), a DNA-binding domain (DBD), a hinge region, and a C-terminal ligand-binding domain (LBD) (52). The NTD is encoded by exon 2 and is the least conserved region of the NR family. It is inherently unstructured, vulnerable to proteases and only becomes structured when the protein binds DNA and forms dimers (70). In the NTD the ligand independent activation function 1 (AF1) is located. This AF1 binds cofactors, chromatin modulators, and the transcription machinery (71–73). The GR DBD is encoded by exons 3 and 4 and is important for DNA binding and GR dimerization. It is characterized by two highly conserved subdomains each containing a Cys4-type zinc finger. In the first subdomain the GR's proximal box (P box) is contained which is important for site specific GR DNA binding. The second subdomain contains the distal box (D box) which is important for GR dimerization (74). Exons 5–9 of the *NR3C1* gene encode the GR's hinge region and LBD. The former provides both flexibility between the DBD and LBD as well as a regulatory interface. The hinge region can be acetylated (lysine residues) and is a target of CLOCK/BMAL acetylation and the presence of acetyl moieties in this area reduces GR activity. Research has also shown that the interaction between the GR and CLOCK/BMAL can be uncoupled, such as by chronic stress or night shift work, which may cause hypercortisolism related pathologies (75, 76). The latter contains a ligand binding pocket, which is formed by 12 α-helices and 4 β-sheets, and the ligand-dependent AF-2 domain. The LBD has also been found important in GR dimerization (77). Further, nuclear localization (NLS), nuclear export (NES), and nuclear retention signals (NRS) have been identified in the GR protein and these are important for the subcellular distribution of the GR. Two NLS have been identified, one in the DBD and one in the LBD (78). A NES is located between the 2 zinc fingers (79) and a NRS delaying GR nuclear export overlaps with NLS1 (80).

Not a single, but multiple GR protein isoforms are identified. This is the result from alternative splicing and the use of 8 different translation initiation start sites (81). Alternative splicing at exon 9 results in two different GR splice variants, namely the classical 777 AA-long GRα or the 742 AA-containing GRβ (8). Both isoforms are identical up to AA 727, but contain non-homologous AA thereafter. Hence, GRβ has a shortened LBD lacking helix 12 and therefore it cannot bind GCs (82). Despite this, GRβ is constitutively found in the nucleus where performs several functions. It was believed and later also shown to be an antagonist to the GRα isoform. Several mechanisms have been proposed for the dominant negative action of GRβ, such as competing with GRα for GR-binding sites and co-regulators and the formation of inactive GRα/β heterodimers (82–84). The role of the GRβ is more extensive than being a simple antagonist. Other studies have shown that the GRβ regulates gene transcription of non-GRα target genes in an GRα and GC independent manner (85). Furthermore, while GRβ cannot bind endogenous GCs, it was show to bind the GR antagonist RU-468, and is modulated by it (86). Perhaps some synthetic GR agonists could also bind to this isoform. The GRβ isoform plays a role in GC resistance (insensitivity to GC treatment) in patients for several diseases. This resistance can be caused by its GRα antagonism as well as by the transcriptome changes its presence causes. A recent study showed that overexpression of GRβ in colonocytes causes dysregulation of many genes also found back in IBD patients (87). Next to GRα and GRβ, GRγ, GR-A, and GR-B splice variants have also been identified (illustrated in Figure 3). All splice-isoforms show diminished activity compared to GRα (88–90). Besides splicing, GR mRNA is further regulated post-transcriptionally via adenine uridylate-rich elements (ARE) in the 3′ UTR of the GR mRNA which mediate GR destabilization (91). Next to this, GR mRNA stability is also regulated by microRNAs (for example: miR-124) which bind to their binding motifs, mostly in the 3′ UTR (92, 93).

Eight GRα translation initiation variants have been identified (GRα-A, -B, -C1, -C2, -C3, -D1, D2, and D3) which is the result from the existence of 8 highly conserved AUG start codons in exon 2 (Figure 3) (94). The AUG start codons are differently selected due to ribosomal leaky scanning and ribosomal shunting mechanisms (94). Because the same AUG start sites are also present in the GR splice-variants, all the translation-initiation isoforms are expected to occur in each of the splice-variants (95). The GR translation variants all have a similar GC and glucocorticoid responsive element (GRE)-binding affinity, but they differ in the length of their N-termini and their transcriptional activity. They show different subcellular localization, regulate distinct sets of genes and their relative levels vary between and within cells (94). The mechanism of regulation of alternative translation start sites and alternative splicing in response to physiological, pathological, and cell-specific signals is still poorly understood. *In vitro* work proved that these isoforms do have the capability to regulate distinct transcriptional programs (96). A later study showed that the different isoforms can regulate apoptosis with the GRα-C3 being pro-apoptotic and the GRα-D3 anti-apoptotic (97).

### GR Activation and Nuclear Translocation

**Figure 4:**
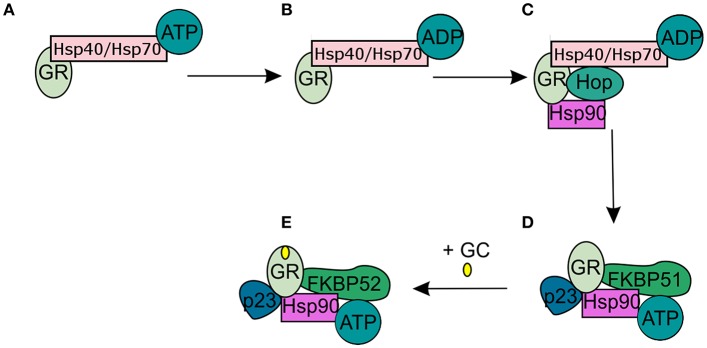
Glucocorticoid receptor chaperone complex and maturation. **(A)** After glucocorticoid receptor (GR) translation an Hsp70-Hsp40-GR complex is formed in the cytoplasm. **(B)** A subsequent ADP-dependent Hsp70 change induces the binding of Hop. **(C)** Hop induces the binding of Hsp90. **(D)** After Hsp90 binding to Hop, Hsp70, and Hsp40 are released from the chaperone complex and replaced by p23 and FKPB51. The GR has now matured into a high affinity complex. **(E)** After binding of glucocorticoids FKBP51 is replaced by FKBP52, which is necessary for the transport of the GR to the nucleus.

In the absence of intracellular bioactive GCs, the GR finds itself as a monomer in the cytoplasm where it resides in a multiprotein complex. This chaperone complex is important for GR maturation, ligand binding, nuclear transport, and activation. The composition of the chaperone complex changes during the different GR maturation/activation states (Figure 4) (98). After GR translation the GR is bound by Hsp70, an interaction that is accelerated by the Hsp40 co-chaperone. Once the folding process is complete GR is transferred from Hsp40/Hsp70 to Hsp90, a transfer that is mediated by Hop (99–101). Recruitment of p23 (102) and FKBP51 to the multiprotein complex leads to maturation of GR-chaperone complex into a conformation that has very high affinity for GR ligands. After GC-binding the GR-chaperone complex again reorganizes (FKBP51 is replaced by FKBP52) and a GR conformational change is induced, leading to the exposure of the GR's 2 nuclear localization signals (103). These are subsequently bound by nucleoporin and importins that carry the GR through the nuclear pore complex into the nucleus (104, 105). Initially it was believed that the GR disassociates from the cytoplasmic chaperone complex upon ligand binding. However, recent research has shown that the chaperone complex is required for efficient nuclear translocation of the receptor (106).

Once inside the nucleus, the activated GR can go on to exert its function or it can be transported back to the cytoplasm, inhibiting the GR's transcriptional activity. Nuclear export of GR is regulated by exportins and calreticulin (CRT) which binds to the GR NES, thereby disrupting the GR-DNA binding (107, 108).

The balance between nuclear import and export determines the proportion of GR protein in the nucleus and has a direct influence on the strength of GR's transcriptional activities. In the nucleus, the GR acts as a TF that can activate (trans-activation) or inhibit (trans-repression) genes as well as modulate the function of other TFs (tethering). Most of the GR functions are restricted to the nucleus, but some non-nuclear actions of GR are also known.

### GR Function

**Figure 5:**
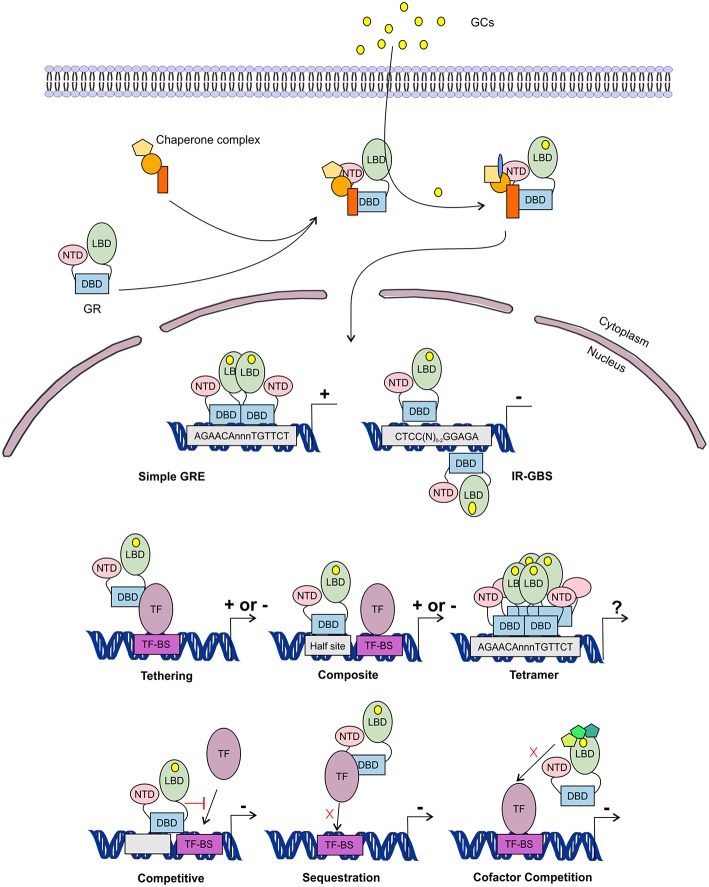
Glucocorticoid receptor activation and function. Lipophilic glucocorticoids (GCs) diffuse through the cell membrane and bind the glucocorticoid receptor (GR) in the cytoplasm. This induces a change in the chaperone complex bound to GR, after which it translocates to the nucleus to transactivate (+) or transrepress (-) gene transcription as a monomer or a dimer. The GR can transactivate genes by binding to glucocorticoid responsive elements (GRE) as a dimer, but also as a monomer by binding to other transcription factors (TF) through tethering or by binding to composite-elements. The GR can further transrepress gene-expression by binding to inverted repeat GR-binding sequences (IR-GBS), by tethering, by composite-elements, by competing for DNA binding-sites (BS), by sequestrating TFs and by competing for cofactors with other TFs. GR might also function as a tetramer, but its function is not known.

In the nucleus, the GR is able to transcriptionally activate (transactivate (TA)) or transcriptionally repress (transrepress (TR)) gene-expression, both as a monomer and as a dimer, and usually via direct contact with DNA. Recently it was discovered that the GR can also bind to the DNA as a tetramer (Figure 5) (109, 110). The importance of this GR tetramer in transcriptional regulation is not well-understood and needs further investigation.

The GR associates with specific genomic loci and orchestrates the assembly of TF regulatory complexes containing the GR, other TFs and co-regulators that modulate the activity of the RNA polymerase II (RNApolII). Different modes of genomic GR transcriptional regulation are described (Figure 5).

**Figure 6:**
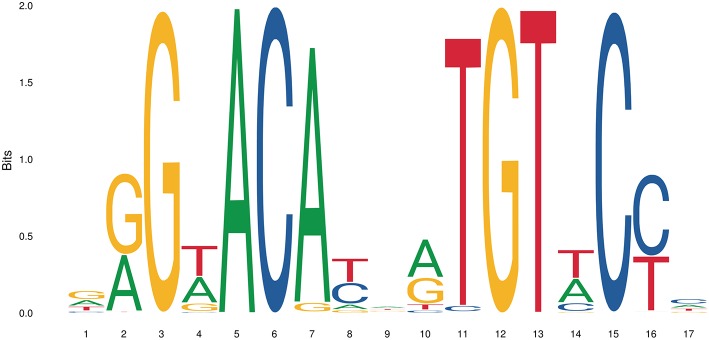
Sequence logo of the human glucocorticoid responsive element to which the GR binds to. See text for more details.

The simplest form of GR-DNA interaction is the binding of GR to genomic glucocorticoid binding sites (GBS) containing a GRE. Classically, the GR exerts its transactivation function by binding to GREs, which are 15 bp long sequence motifs of 2 imperfect inverted palindromic repeats of 6 bp separated by a 3 bp spacer. The generally accepted GRE consensus sequence is AGAACAnnnTGTTCT. However, this may be better represented as a sequence logo (Figure 6), which illustrates that some positions are much more variable than others. The GR binds to the GRE as a homodimer and each GR DBD makes contact with about 3 nucleotides in each of the half site hexamers. The two GR molecules bind the GRE in a head-to-tail fashion and 5 AA within the D box of the second GR zinc finger provide critical protein-protein contacts between the two GR partners important for stabilization of the GR DBD on the DNA. In this D box a hydrogen bond is formed between Ala458 of one dimer partner and Ile483 of the other partner (74, 111). A second interface important for dimerization (Ile628) has been identified in the LBD (77, 112). Recent research proposes that the LBD may have other dimerization interfaces related to another dimer structure (113).

GREs contain relatively few highly conserved residues and because GREs are rather short, they are abundantly present in the genome. ChIP-seq experiments with antibodies against GR showed however that only a small fraction of GRE sites are in fact occupied by the GR (114). Why this is the case is still a topic of research, but it has been shown that the chromatin structure plays a big role in determining which sites are accessible to GR under certain conditions (115, 116). It has also been shown that many GR binding sites can be found very far from a (known) gene or transcriptionally active sites, indicating that GR often occupies enhancer regions and/or chromatin looping is involved in GR transcriptional regulation (114).

Evidence has been found for a 2nd mode of GR-DNA interaction where GR, as a monomer, binds to half sites with an AGAACA (or the reverse complement TGTTCT) consensus sequence (117). If a binding site for another TF is nearby the GRE-half site, both elements may act as a composite site where there is an interaction (positive or negative) between the GR (monomer) and the other TF (118) (Figure 5). An analysis in mouse liver showed that under endogenous corticosterone levels (i.e., low concentrations) GR binding to half sites as a monomer is more prevalent than binding of full GRE sites by homodimers. In response to exogenous GCs (i.e., high concentration) the GR dimers assemble on full length GRE near known induced genes and this happens in concert with monomer removal of sites near repressed genes (119).

A third class of GR-DNA interactions involves inverted-repeat GBS (Figure 5). Binding to such an element leads to inhibition of gene expression. These IR-nGREs have a consensus CTCC(N)_0−2_GGAGA sequence and structural analysis showed that at these sites 2 GR monomers bind on the opposite sides of the DNA, in a head-to-tail orientation and with negative co-operativity with each other (120, 121).

Lastly, there are the indirect binding, or tethering, sites where GR is recruited to a TF complex through protein-protein interactions with heterologous DNA-bound TFs (Figure 5). These GBSs lack a GRE, IR-nGRE, or a GRE half site. Several TFs are known to recruit ligand bound GR via tethering including members from the AP1, STAT, and NF-κB families of TFs. These interactions directly alter the capacity of the directly DNA-bound TF to bind DNA, recruit cofactors, and activate/repress gene transcription (122, 123).

The GR can also TR gene-expression by competing with other TFs for binding to overlapping DNA-binding sequences. Indeed, recently GR half-sites were even found embedded in AP-1 response elements (124). Finally, the GR can TR gene-expression by competing with other TFs for the binding of cofactors (125–127) or by sequestrating TFs, thereby obstructing them to bind to the DNA (128) (Figure 5).

### GR Plasticity

The GR operates in a cell- and context-specific manner. This is not only due to a different expression of GR protein isoforms but is also the cause of different signals that modulate the GR's activity at specific GBSs. Four signals are described to influence the GR's function.

A first signal that modulates GR activity is the DNA, which acts as an allosteric regulator of the GR. GRE sequences differing by only one single base pair were namely shown to affect GR conformation and regulatory activity (129). Moreover, allosteric changes provoked by one half site can be transduced via the GR lever arm (located between the P and D box, see Figure 3) and the receptor's D box to the dimer partner, affecting the GR's transcriptional activity (130, 131).

A second signal influencing the GR transcriptional output obviously comes from the ligand that binds to the LBD. After ligand-binding helix 12 is exposed and cofactors are recruited to the AF2 in the LBD. Depending on the ligand, the LBD will adopt another conformation and attract other cofactors thereby influencing the GR's transcriptional outcome (132, 133). The latter forms the basis of the research for “Selective GR Agonists and Modulators” (SEGRAM).

Third, the GR is heavily modified by potential post-translational modifications (PTMs). Several phosphorylation (134–140), ubiquitination (141), sumoylation (142), acetylation (76), and nitrosylation sites (143) as depicted in Figure 3 have been identified influencing GR-localization, stability, DNA binding, ligand response, and regulatory activity.

Last, the GR's transcriptional output is influenced by its interaction partners. These include other TFs that bind direct or indirect to GR and cofactors which are recruited to GR and are involved in functions such as chromatin regulation and regulation of the transcriptional machinery function (53, 144). The composition of the cofactor complex recruited to the GR depends on the cell specific expression of cofactors, the cell context and the integration of the previous described signals (DNA, ligand, and PTMs) that influence the GR's conformation (145). This cofactor complex eventually determines the transcriptional output of the GR.

### Non-genomic GC and GR Actions

The GR is not only able to function by genomic actions, but also through non-genomic actions. Non-genomic GC/GR actions are fast and do not require transcription or protein synthesis. Limited knowledge is however available on non-genomic GC/GR actions. These include GC-mediated effects on membrane lipids, changing their physicochemical properties (146). Further, GCs have also been seen to act on a membrane-bound GR which is related to the classical GR and probably the result from differential splicing, alternative transcription initiation and PTMs (146, 147). Another membrane receptor, unrelated to the classical GR, probably also binds GCs. This protein is probably a G-coupled receptor that signals through cAMP and that binds endogenous GCs with high affinity. However, it does not bind most GC analogs such as dexamethasone (148). Other non-genomic actions, e.g., modulation of the MAPK signaling cascade, might result from components that are released from the GR chaperone complex upon the binding of GCs to the GR or from membrane bound GR (149, 150).

A final type of non-genomic action of the GR is its effect on mitochondrial function. It was show that the GR can translocate to and reside in mitochondria (151, 152). This mitochondrial GR is capable of regulating gene transcription from the mitochondrial chromosome by binding to GRE like elements alone or in complex with other factors. This was demonstrated *in vitro*, using a hepatoma cell line and in brain cell of mice and rats (153–155). A recent study showed that a GR isoform, GR?, is located in the mitochondria and plays a role in regulating cell energy metabolism in a ligand independent manner (156).

## GC Therapy: Drawbacks and Optimization

GCs are therapeutically mainly used for their anti-inflammatory and immunosuppressive effects. These are a.o. the result of the transcriptional induction of several anti-inflammatory protein-coding genes such as *TSC22D3* (coding for glucocorticoid-induced leucine zipper, GILZ) and *DUSP1* (coding for Map Kinase Phosphatase 1, MKP1) and from the repression of pro-inflammatory TFs such as NF-κB and AP-1. GCs are used to treat inflammatory disorders such as asthma (157), skin rashes (158), rheumatoid arthritis (RA) (159), multiple sclerosis (160), and systemic lupus erythematosus (SLE) (161). In most cases, synthetic glucocorticoids are used but hydrocortisone is also a popular option.

**Figure 7:**
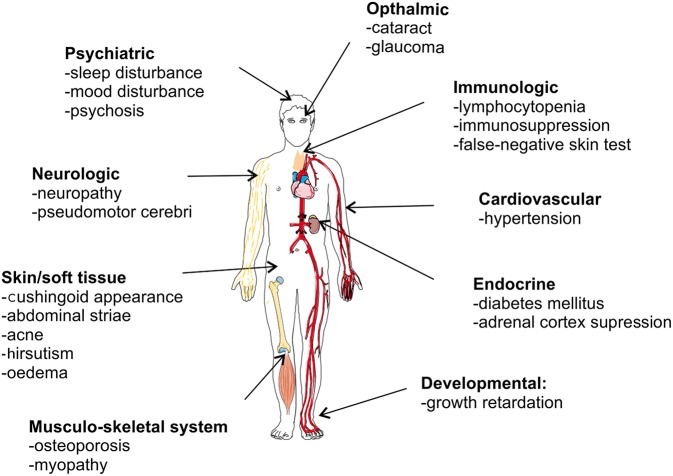
Overview of glucocorticoid-associated side effects.

Despite its strong anti-inflammatory capacity, GC therapy is limited by two major drawbacks. First, GCs are well-known to be associated with adverse effects, particularly when given in high doses for long time periods. Figure 7 graphically presents GC-associated side effects, with osteoporosis, hyperglycemia, cardiovascular diseases, and infections as the four most worrisome adverse effects for clinicians (162). These side effect may be severe enough to affect the therapy or cause an increased risk to other negative effects. A recent study in RA patients showed a clearly increased risk of bone fractures correlated with the administration of GCs (osteoporosis) (163). Second, some patients are refractory to the therapy and are GC resistant (GCR). GCR can either be inherited, mostly via mutations in the *NR3C1* gene (52, 164), or acquired (165). The latter can be caused by ligand induced homologous downregulation of the GR, caused chronical GC treatment (166, 167), or by pathophysiological processes accompanying the inflammatory disease states [e.g., chronic obstructive pulmonary disease (COPD) (168), SLE (169)]. The pathophysiological processes provoking GCR are very heterogeneous, e.g., oxidative stress and inflammatory cytokines are known triggers of GCR and have multiple effects on GR biology (170–176). GCR occurs in 4–10% of the asthma patients, 30% of the RA patients and in almost all of the sepsis and COPD patients (177–179).

To achieve a positive benefit-to-risk ratio when using GCs, guideline recommendations regarding optimal dosing must be followed and potential adverse effects must be monitored, prevented and managed (180–183). Next to this, much research effort is put in developing innovative GCs or GR ligands that improve the therapeutic balance (184–186).

Currently available GCs in the clinic activate all GR activities. During the past 20 years intensive research for SEGRAMs, which promote a GR conformation favoring TR over TA, has been performed. This search for SEGRAMs is based on the central dogma in GR biology which states that GR monomer-mediated TR is sufficient to counteract inflammation, while GR dimer-mediated TA is responsible for most of the adverse effects of GCs, e.g., by the induction of genes encoding glucose-6-phosphatase (*G6P*) and phosphoenolpyruvate carboxykinase (*PCK1*). This long accepted dogma in GR biology originates from initial work with the GR^Dim^ mutant (187). This GR^Dim^ mutant carries a A465T mutation in the D-loop of the second zinc finger of the GR-DBD.

This D-loop is one of the primary dimerization interfaces, consequently this mutant shows impaired homodimerization and reduced functionality. Initial observation on the GR^Dim^ mutant showed a strongly impaired transactivation and retained capability to transcriptionally repress genes, particularly as a monomer (111). Follow-up work on the GR^Dim^ found that there was still transactivation of certain genes possible by these mutant receptors (129, 131). This raised the question again if the GR^Dim^ was still capable of some dimerization and or DNA binding. An *in vivo* imaging study with labeled GR showed that the GR^Dim^ is still capable of dimerization with endogenous and synthetic GCs, but with a lower efficiency than WT for endogenous GCs (188). The ability of the GR^Dim^ mutant to bind to the DNA has been a point of controversy since there is evidence against (111, 189) and pro DNA binding (131, 190, 191). Current evidence seems to suggest that the DNA binding capacity of the mutant is at least partially preserved. A second GR mutant was generated with an additional point mutation in the LBD of the receptor. This mutation is believed to disrupt a secondary dimerization interface present in the LBD, leading to even poorer dimerization and function than the GR^Dim^ mutant (188). In addition, under normal physiological conditions, GR^Dim^ mice are healthy and show no obvious phenotypes, except that they express interferon genes in their intestinal epithelium (192), It has been shown that under physiological conditions, GR binds to the DNA as a monomer, exerting transcriptional functions related to cell-type-specific functions, and that only after acute stress or injection of GCs, GR dimers are formed leading to binding to full GRE elements (119). Also, elegant, NMR-based work by Watson et al. has shown that, depending on the DNA sequence where GR dimers bind, an intramolecular signal, via a lever arm, provides a dimer- and DNA-binding-stabilizing interaction between two DBD domains, precisely via the amino acid that was mutated in the GRDim version. The absence of this amino acid “weak binding” in the GR^Dim^ version was enough to cause less robust dimers and DNA binding (131).

It has been stated that the picture about the mechanisms of glucocorticoid actions (transactivation/transrepression) is still far from complete, especially for known GR mutants. In addition, the aforementioned functional PPI interfaces, recent structural biology work shows that the knowledge on GR dimerization and structural conformation may be incomplete based on structural homology and residue conservation between the NR transcription factor family, and new dimer interfaces that remain unexplored so far. In one study researchers have postulated that the conformation of the GR that is generally accepted as the dimeric conformation might not be correct and they propose different configurations (113). The fact most of the structural work so far was done on subdomains of the GR, as the whole protein is very hard to crystalize, may contribute to this limited knowledge of GR structure.

Many studies have investigated steroidal and non-steroidal SEGRAM in the hope to be able to dissociate the GC-induced anti-inflammatory effects from the GC-induced side effects (193–197). Several interesting SEGRAM have been characterized [e.g., Al-438, LGD-5552, ZK216348, Mapracorat and Compound A (CpdA)] and were shown to have dissociative profiles *in vivo* (198–206). Despite the intensive research, none of the SEGRAM have reached the market today. So far, only Fosdagrocorat (for RA) (207–209) and Mapracorat (for ocular inflammatory diseases and skin inflammation) have reached clinical trials.

To prevent GC-induced side effects, strategies other than shifting the balance between the monomeric and the dimeric GR are also followed (184–186). Some aim at cell-specific targeting of GCs via antibody- or peptide-GC conjugates (210) or via liposomes (211), thereby preventing systemic GC-effects. Other studies investigate the therapeutic use of GC-induced proteins (e.g., GILZ, the protein coded by the *TSC22D3* gene) without administrating GCs themselves. By this, steps are undertaken to develop therapies that stimulate only the wanted anti-inflammatory GC-functions without inducing the broad and also the unwanted GC-effects (212). Further, studies also invest in the therapeutic potential of combination therapies, such as the combination of GR and PPAR agonists (213, 214).

## GC Therapy in Acute vs. Chronic Inflammation: Sirs and the Sedigram Concept

During the recent years, it has become clear that the old idea in GC-research, that claims that GC anti-inflammatory effects can be separated from GC-induced side effects by simply dissociating GR TR from GR TA, because the former would be mainly monomeric-driven GR functions and the latter GR homodimeric-driven functions. To date it is known that this separation cannot be made that strictly. In addition, GR^Dim^ mice studies showed that not all GC-induced side effects are GR dimer-driven and that thus also monomeric GR is involved in at least some side effects. Indeed, GR^Dim^ mice were observed to develop osteoporosis and muscle atrophy, despite their lack of GR dimer-dependent effects (215, 216). Next to this, the GR dimer was found to be indispensable for the GC-mediated protection in models of acute inflammation. GR^Dim^ mice are strongly sensitized in models of TNF- and LPS-induced Systemic Inflammatory Response Syndrome (SIRS) (217, 218) and these mice could furthermore no longer be protected by a prophylactic Dexamethasone administration (192). Additionally, GR dimer-induced GRE genes were found to be important in the protection against SIRS: this was shown for *DUSP1* (217) (encoding MKP-1) and *TSC22D3* (212) (encoding GILZ). Finally, skewing the GR toward the monomer by using CpdA sensitized mice for TNF-induced SIRS, suggesting that GR monomers are unable to protect in this model of acute inflammation and that GR monomers should rather be avoided in SIRS (219). Altogether these data illustrate the importance of the GR dimer in the protection against acute-inflammation.

As a consequence of the former observations in GR^Dim^ mice, the SEGRAM concept needed to be revised. Therefore, recently, it was proposed that chronic inflammatory diseases which require a long-term GC therapy would benefit from “Selective Monomer GR Agonists and Modulators” (SEMOGRAMs), since these SEMOGRAMs would avoid important side effects such as hyperglycemia that are detrimental for the patients. Recently, it was also observed that ligand-induced GR turnover leading to GCR is GR dimer dependent (220). The latter observation thus further supports the need for SEMOGRAMs for the treatment of chronic inflammation. On the other hand, in acute-inflammatory settings such as SIRS, where GR dimers are indispensable, the administration of GCs that increase the GR dimerizing potential, termed “Selective Dimer GR Agonists and Modulators” (SEDIGRAMs), would be the preferred strategy to follow (221).

There has been some doubt about the value of the GR^Dim^ mouse tool and its inability to form homodimers and bind DNA. Although *in vitro* experiments (making use of high GC-doses) showed very little effect of the Dim-mutation on GR dimerization and DNA binding (188, 191), *in vivo* research confirmed that GC-induced transcription is very broadly hampered in GR^Dim^ vs. GR^WT^ mice (192, 222). Moreover, the remaining GR^Dim^ transcription was observed to be especially the result of GR monomer functioning at half-sites (119). Although we are aware that a second interface in the GR LBD is also of relevance for dimerization and that remaining dimerization in the GR^Dim^ mutant is probably provided through this protein-protein contact, the latter studies confirm the value of the GR^Dim^ mouse-tool.

## Future Perspectives

Certain challenges and (new) questions remain to be answered or further investigated. GCR in patients is still largely an unresolved issue, especially in complex diseases such as sepsis but also in severe asthma. Understanding GC resistance, preventing or reverting it could mean a real breakthrough in current medical practice. Another avenue of research, aside from more selective dimer/monomer ligands, is GR structure and DNA binding conformation as some more recent research suggested that the GR can bind to DNA is a tetramer conformation instead of a dimer. Also, the non-genomic effects of GCs and GR are far from understood and need more research. Finally, a wealth of information has been published using a variety of GR ligands, some being endogenous ligands, others synthetic ligands, all of which may have very different effects on the canonical GR and non-canonical ones (splice variants, shorter proteins) and even different effects in different mammalian species or cell types. It is a big challenge for the community to try to streamline this information in a comprehensive way.

## Author Contributions

JS and ST wrote the manuscript. CL reviewed the manuscript.

### Conflict of Interest Statement

The authors declare that the research was conducted in the absence of any commercial or financial relationships that could be construed as a potential conflict of interest.

## References

[1] Ten S New M Maclaren N. Clinical review 130: Addison's disease 2001. J Clin Endocrinol Metab. (2001) 86:2909–22. 10.1210/jcem.86.7.7636 11443143

[2] Simoni RD Hill RL Vaughan M The isolation of thyroxine and cortisone: the work of Edward C. Kendall. J Biol Chem. (2002) 277:e10 Retrieved from: http://www.jbc.org/content/277/21/e10.full

[3] Sarett LH Partial synthesis of pregnene-4-triol-17(b), 20(b), 21-dione-3, 11 and pregnene-4-diol-17(b),21-trione-3,11,20 monoacetate. J Biol Chem. (1946) 162:601–32. 21018769

[4] Hench PS Kendall EC Slocumb CH Polley HF. The effect of a hormone of the adrenal cortex (17-hydroxy-11-dehydrocorticosterone; compound E) and of pituitary adrenocorticotropic hormone on rheumatoid arthritis. Proc Staff Meet Mayo Clin. (1949) 24:181–97. 18118071

[5] Germain P Staels B Dacquet C Spedding M Laudet V. Overview of nomenclature of nuclear receptors. Pharmacol Rev. (2006) 58:685–704. 10.1124/pr.58.4.2 17132848

[6] Gustafsson JA. Historical overview of nuclear receptors. J Steroid Biochem Mol Biol. (2016) 157:3–6. 10.1016/j.jsbmb.2015.03.004 25797032

[7] Munck A Brinck-Johnsen T. Specific and nonspecific physicochemical interactions of glucocorticoids and related steroids with rat thymus cells *in vitro*. J Biol Chem. (1968) 243:5556–65. 5699052

[8] Hollenberg SM Weinberger C Ong ES Cerelli G Oro A Lebo R. Primary structure and expression of a functional human glucocorticoid receptor cDNA. Nature. (1985) 318:635–41. 10.1038/318635a0 2867473 PMC6165583

[9] Weinberger C Hollenberg SM Ong ES Harmon JM Brower ST Cidlowski J. Identification of human glucocorticoid receptor complementary DNA clones by epitope selection. Science. (1985) 228:740–2. 10.1126/science.2581314 2581314

[10] Reul JM de Kloet ER. Two receptor systems for corticosterone in rat brain: microdistribution and differential occupation. Endocrinology. (1985) 117:2505–11. 10.1210/endo-117-6-2505 2998738

[11] Chrousos GP Kino T Glucocorticoid action networks and complex psychiatric and/or somatic disorders. Stress. (2007) 10:213–9. 10.1080/10253890701292119 17514590

[12] Vegiopoulos A Herzig S. Glucocorticoids, metabolism and metabolic diseases. Mol. Cell. Endocrinol. (2007) 275:43–61. 10.1016/j.mce.2007.05.015 17624658

[13] Hawkins UA Gomez-Sanchez EP Gomez-Sanchez CM Gomez-Sanchez CE. The ubiquitous mineralocorticoid receptor: clinical implications. Curr Hypertens Rep. (2012) 14:573–80. 10.1007/s11906-012-0297-0 22843494 PMC3491176

[14] Cruz-Topete D Cidlowski JA. One hormone, two actions: anti- and pro-inflammatory effects of glucocorticoids. Neuroimmunomodulation. (2014) 22:20–32. 10.1159/000362724 25227506 PMC4243162

[15] Bosscher K Haegeman G. Minireview: latest perspectives on antiinflammatory actions of glucocorticoids. Mol Endocrinol. (2008) 23:281–91. 10.1210/me.2008-0283 19095768 PMC5428155

[16] Donatti T Koch V Takayama L Pereira R. Effects of glucocorticoids on growth and bone mineralization. J Pediatria. (2011) 87:4–12. 10.2223/JPED.2052 21234507

[17] Nussinovitch U de Carvalho JF Pereira RM Shoenfeld Y. Glucocorticoids and the cardiovascular system: state of the art. Curr Pharm Des. (2010) 16:3574–85. 10.2174/138161210793797870 20977421

[18] Cruz-Topete D Myers PH Foley JF Willis MS Cidlowski JA. Corticosteroids are essential for maintaining cardiovascular function in male mice. Endocrinology. (2016) 157:2759–71. 10.1210/en.2015-1604 27219275 PMC4929548

[19] Farrell C O'Keane V. Epigenetics and the glucocorticoid receptor: a review of the implications in depression. Psychiatry Res. (2016) 242:349–56. 10.1016/j.psychres.2016.06.022 27344028

[20] Joëls M. Impact of glucocorticoids on brain function: relevance for mood disorders. Psychoneuroendocrinology. (2011) 36:406–14. 10.1016/j.psyneuen.2010.03.004 20382481

[21] Tatomir A Micu C Crivii C. The impact of stress and glucocorticoids on memory. Clujul Med. (2014) 87:3–6. 10.15386/cjm.2014.8872.871.at1cm2 26527987 PMC4462413

[22] Whirledge S Cidlowski JA. Glucocorticoids and reproduction: traffic control on the road to reproduction. Trends Endocrinol Metab. (2017) 28:399–415. 10.1016/j.tem.2017.02.005 28274682 PMC5438761

[23] Fowden AL Forhead AJ. Glucocorticoids as regulatory signals during intrauterine development. Exp Physiol. (2015) 100:1477–87. 10.1113/EP085212 26040783

[24] Miller WL. Androgen synthesis in adrenarche. Rev Endocr Metab Disord. (2009) 10:3–17. 10.1007/s11154-008-9102-4 18821018

[25] Talaber G Jondal M Okret S. Local glucocorticoid production in the thymus. Steroids. (2015) 103:58–63. 10.1016/j.steroids.2015.06.010 26102271

[26] Noti M Sidler D Brunner T. Extra-adrenal glucocorticoid synthesis in the intestinal epithelium: more than a drop in the ocean? Semin Immunopathol. (2009) 31:237–48. 10.1007/s00281-009-0159-2 19495759

[27] Jozic I Stojadinovic O Kirsner RS Tomic-Canic M Stressing the steroids in skin: paradox or fine-tuning? J Invest Dermatol. (2014) 134:2869–72. 10.1038/jid.2014.363 25381768

[28] Taves MD Gomez-Sanchez CE Soma KK. Extra-adrenal glucocorticoids and mineralocorticoids: evidence for local synthesis, regulation, and function. Am J Physiol Endocrinol Metab. (2011) 301:E11–24. 10.1152/ajpendo.00100.2011 21540450 PMC3275156

[29] Mittelstadt PR Taves MD Ashwell JD. Cutting edge *de novo* glucocorticoid synthesis by thymic epithelial cells regulates antigen-specific thymocyte selection. J Immunol. (2018) 200:1988–94. 10.4049/jimmunol.1701328 29440508 PMC5840004

[30] Huang J Jia R Brunner T. Local synthesis of immunosuppressive glucocorticoids in the intestinal epithelium regulates anti-viral immune responses. Cell Immunol. (2018) 334:1–10. 10.1016/j.cellimm.2018.08.009 30144940

[31] Spiga F Walker JJ Terry JR Lightman SL. HPA axis-rhythms. Compr Physiol. (2014) 4:1273–98. 10.1002/cphy.c140003 24944037

[32] Malkoski SP Dorin RI. Composite glucocorticoid regulation at a functionally defined negative glucocorticoid response element of the human corticotropin-releasing hormone gene. Mol Endocrinol. (1999) 13:1629–44. 10.1210/mend.13.10.0351 10517666

[33] Drouin J Lin S Nemer M. Glucocorticoid repression of pro-opiomelanocortin gene transcription. J Steroid Biochem. (1989) 34:63–9. 10.1016/0022-4731(89)90066-6 2626052

[34] De Kloet R Vreugdenhil E Oitzl MS Joëls M. Brain corticosteroid receptor balance in health and disease. Endocrine Rev. (1998) 19:269–301. 10.1210/er.19.3.269 9626555

[35] Martens C Bilodeau S Maira M Gauthier Y Drouin J. Protein-protein interactions and transcriptional antagonism between the subfamily of NGFI-B/Nur77 orphan nuclear receptors and glucocorticoid receptor. Mol Endocrinol. (2005) 19:885–97. 10.1210/me.2004-0333 15591535

[36] Bilodeau S Vallette-Kasic S Gauthier Y Figarella-Branger D Brue T Berthelet F. Role of Brg1 and HDAC2 in GR trans-repression of the pituitary POMC gene and misexpression in Cushing disease. Genes Dev. (2006) 20:2871–86. 10.1101/gad.1444606 17043312 PMC1619949

[37] Di S Malcher-Lopes R Halmos K Tasker JG. Nongenomic glucocorticoid inhibition via endocannabinoid release in the hypothalamus: a fast feedback mechanism. J Neurosci. (2003) 23:4850–7. 10.1523/JNEUROSCI.23-12-04850.2003 12832507 PMC6741208

[38] Di S Maxson MM Franco A Tasker JG. Glucocorticoids regulate glutamate and GABA synapse-specific retrograde transmission via divergent nongenomic signaling pathways. J Neurosci. (2009) 29:393–401. 10.1523/JNEUROSCI.4546-08.2009 19144839 PMC2775558

[39] Hammond GL. Plasma steroid-binding proteins: primary gatekeepers of steroid hormone action. J Endocrinol. (2016) 230:R13–25. 10.1530/JOE-16-0070 27113851 PMC5064763

[40] Hammond GL Ith CL Paterson NA Sibld WJ. A role for corticosteroid-binding globulin in delivery of cortisol to activated neutrophils^*^. J Clin Endocrinol Metab. (1990) 71:34–9. 10.1210/jcem-71-1-34 2370299

[41] Seckl JR. 11beta-hydroxysteroid dehydrogenases: changing glucocorticoid action. Curr Opin Pharmacol. (2004) 4:597–602. 10.1016/j.coph.2004.09.001 15525550

[42] Draper N Stewart PM. 11beta-hydroxysteroid dehydrogenase and the pre-receptor regulation of corticosteroid hormone action. J Endocrinol. (2005) 186:251–71. 10.1677/joe.1.06019 16079253

[43] Yeager MP Pioli PA Guyre PM. Cortisol exerts bi-phasic regulation of inflammation in humans. Dose Response. (2011) 9:332–47. 10.2203/dose-response.10-013.Yeager 22013396 PMC3186928

[44] O'Connor KA Johnson JD Hansen MK Frank JLW Maksimova E Watkins LR. Peripheral and central proinflammatory cytokine response to a severe acute stressor. Brain Res. (2003) 991:123–32. 10.1016/j.brainres.2003.08.006 14575884

[45] Dhabhar FS McEwen BS. Enhancing versus suppressive effects of stress hormones on skin immune function. Proc Natl Acad Sci USA. (1999) 96:1059–64. 10.1073/pnas.96.3.1059 9927693 PMC15350

[46] Buchwald P Bodor N. Soft glucocorticoid design: structural elements and physicochemical parameters determining receptor-binding affinity. Die Pharmazie. (2004) 59:396–404. 15212309

[47] Daley-Yates PT. Inhaled corticosteroids: potency, dose equivalence and therapeutic index. Br J Clin Pharmacol. (2015) 80:372–80. 10.1111/bcp.12637 25808113 PMC4574823

[48] Chapman KE Coutinho AE Zhang ZG Kipari T Savill JS Seckl JR Changing glucocorticoid action: 11 beta-Hydroxysteroid dehydrogenase type 1 in acute and chronic inflammation. J Steroid Biochem Mol Biol. (2013) 137:82–92. 10.1016/j.jsbmb.2013.02.002 23435016 PMC3925798

[49] Oprea A Bonnet NCG Polle O Lysy PA. Novel insights into glucocorticoid replacement therapy for pediatric and adult adrenal insufficiency. Ther Adv Endocrinol Metab. (2019) 10:2042018818821294. 10.1177/2042018818821294 30746120 PMC6360643

[50] Diederich S Eigendorff E Burkhardt P Quinkler M Bumke-Vogt C Rochel M 11 beta-hydroxysteroid dehydrogenase types 1 and 2: an important pharmacokinetic determinant for the activity of synthetic mineralo- and glucocorticoids. J Clin Endocrinol Metab. (2002) 87:5695–701. 10.1210/jc.2002-020970 12466373

[51] Huang P Chandra V Rastinejad F. Structural overview of the nuclear receptor superfamily: insights into physiology and therapeutics. Ann Rev Physiol. (2010) 72:247–72. 10.1146/annurev-physiol-021909-135917 20148675 PMC3677810

[52] Vandevyver S Dejager L Libert C. Comprehensive overview of the structure and regulation of the glucocorticoid receptor. Endocr Rev. (2014) 35:671–93. 10.1210/er.2014-1010 24937701

[53] Weikum ER Knuesel MT Ortlund EA Yamamoto KR. Glucocorticoid receptor control of transcription: precision and plasticity via allostery. Nat Rev Mol Cell Biol. (2017) 18:159–74. 10.1038/nrm.2016.152 28053348 PMC6257982

[54] Scheschowitsch K Leite J Assreuy J. New insights in glucocorticoid receptor signaling—more than just a ligand-binding receptor. Front Endocrinol. (2017) 8:16. 10.3389/fendo.2017.00016 28220107 PMC5292432

[55] Galon J Franchimont D Hiroi N Frey G Boettner A Ehrhart-Bornstein M. Gene profiling reveals unknown enhancing and suppressive actions of glucocorticoids on immune cells. FASEB J. (2002) 16:61–71. 10.1096/fj.01-0245com 11772937

[56] Zong J Ashraf J Thompson EB. The promoter and first, untranslated exon of the human glucocorticoid receptor gene are GC rich but lack consensus glucocorticoid receptor element sites. Mol Cell Biol. (1990) 10:5580–5. 10.1128/MCB.10.10.5580 2398904 PMC361279

[57] Breslin MB Geng C-D Vedeckis WV. Multiple promoters exist in the human GR gene, one of which is activated by glucocorticoids. Mol Endocrinol. (2001) 15:1381–95. 10.1210/mend.15.8.0696 11463861

[58] Turner JD Muller CP. Structure of the glucocorticoid receptor (NR3C1) gene 5′ untranslated region: identification, and tissue distribution of multiple new human exon 1. J Mol Endocrinol. (2005) 35:283–92. 10.1677/jme.1.01822 16216909

[59] Presul E Schmidt S Kofler R Helmberg A. Identification, tissue expression, and glucocorticoid responsiveness of alternative first exons of the human glucocorticoid receptor. J Mol Endocrinol. (2007) 38:79–90. 10.1677/jme.1.02183 17242171

[60] Bockmühl Y Murgatroyd CA Kuczynska A Adcock IM Almeida OFX Spengler D. Differential regulation and function of 5'-untranslated GR-exon 1 transcripts. Mol Endocrinol. (2011) 25:1100–10. 10.1210/me.2010-0436 21527501 PMC5417247

[61] Breslin MB Vedeckis WV. The glucocorticoid receptor and c-jun promoters contain AP-1 sites that bind different AP-1 transcription factors. Endocrine. (1996) 5:15–22. 10.1007/BF02738651 21153089

[62] Nunez SB Geng C-D Pedersen K Millro-Macklin CD Vedeckis WV. Interaction between the interferon signaling pathway and the human glucocorticoid receptor gene 1A promoter. Endocrinology. (2004) 146:1449–57. 10.1210/en.2004-0672 15576464

[63] Burnstein KL Jewell CM Cidlowski JA. Human glucocorticoid receptor cDNA contains sequences sufficient for receptor down-regulation. J Biol Chem. (1990) 265:7284–91. 1692020

[64] Radtke KM Schauer M Gunter HM Ruf-Leuschner M Sill J Meyer A. Epigenetic modifications of the glucocorticoid receptor gene are associated with the vulnerability to psychopathology in childhood maltreatment. Transl Psychiatry. (2015) 5:e571 10.1038/tp.2015.63 26080088 PMC4471294

[65] Tyrka AR Price LH Marsit C Walters OC Carpenter LL. Childhood adversity and epigenetic modulation of the leukocyte glucocorticoid receptor: preliminary findings in healthy adults. PLoS ONE. (2012) 7:e30148. 10.1371/journal.pone.0030148 22295073 PMC3266256

[66] Argentieri MA Nagarajan S Seddighzadeh B Baccarelli AA Shields AE. Epigenetic pathways in human disease: the impact of DNA methylation on stress-related pathogenesis and current challenges in biomarker development. EBioMedicine. (2017) 18:327–50. 10.1016/j.ebiom.2017.03.044 28434943 PMC5405197

[67] Sanchez-Vega B Gandhi V. Glucocorticoid resistance in a multiple myeloma cell line is regulated by a transcription elongation block in the glucocorticoid receptor gene (NR3C1). Br J Haematol. (2009) 144:856–64. 10.1111/j.1365-2141.2008.07549.x 19133980 PMC4303606

[68] Nesset KA Perri AM Mueller CR. Frequent promoter hypermethylation and expression reduction of the glucocorticoid receptor gene in breast tumors. Epigenetics. (2014) 9:851–9. 10.4161/epi.28484 24622770 PMC4065183

[69] Kay P Schlossmacher G Matthews L Sommer P Singh D White A. Loss of glucocorticoid receptor expression by DNA methylation prevents glucocorticoid induced apoptosis in human small cell lung cancer cells. PLoS ONE. (2011) 6:e24839. 10.1371/journal.pone.0024839 21984896 PMC3184945

[70] Kumar R Thompson EB. Folding of the glucocorticoid receptor N-terminal transactivation function: dynamics and regulation. Mol Cell Endocrinol. (2012) 348:450–6. 10.1016/j.mce.2011.03.024 21501657

[71] Almlöf T Wallberg AE Gustafsson JA Wright AP. Role of important hydrophobic amino acids in the interaction between the glucocorticoid receptor tau 1-core activation domain and target factors. Biochemistry. (1998) 37:9586–94. 10.1021/bi973029x 9649342

[72] Khan SH Awasthi S Guo C Goswami D Ling J Griffin PR. Binding of the N-terminal region of coactivator TIF2 to the intrinsically disordered AF1 domain of the glucocorticoid receptor is accompanied by conformational reorganizations. J Biol Chem. (2012) 287:44546–60. 10.1074/jbc.M112.411330 23132854 PMC3531768

[73] Kumar R Volk DE Li J Lee JC Gorenstein DG Thompson BE. TATA box binding protein induces structure in the recombinant glucocorticoid receptor AF1 domain. Proc Natl Acad Sci USA. (2004) 101:16425–30. 10.1073/pnas.0407160101 15545613 PMC534534

[74] Luisi BF Xu WX Otwinowski Z Freedman LP Yamamoto KR Sigler PB. Crystallographic analysis of the interaction of the glucocorticoid receptor with DNA. Nature. (1991) 352:497–505. 10.1038/352497a0 1865905

[75] Kino T Chrousos GP. Acetylation-mediated epigenetic regulation of glucocorticoid receptor activity: circadian rhythm-associated alterations of glucocorticoid actions in target tissues. Mol Cell Endocrinol. (2011) 336:23–30. 10.1016/j.mce.2010.12.001 21146585 PMC3057275

[76] Nader N Chrousos GP Kino T. Circadian rhythm transcription factor CLOCK regulates the transcriptional activity of the glucocorticoid receptor by acetylating its hinge region lysine cluster: potential physiological implications. FASEB J. (2009) 23:1572–83. 10.1096/fj.08-117697 19141540 PMC2669420

[77] Bledsoe RK Montana VG Stanley TB Delves CJ Apolito CJ McKee DD. Crystal structure of the glucocorticoid receptor ligand binding domain reveals a novel mode of receptor dimerization and coactivator recognition. Cell. (2002) 110:93–105. 10.1016/S0092-8674(02)00817-6 12151000

[78] Tang Y Getzenberg RH Vietmeier BN Stallcup MR Eggert M Renkawitz R. The DNA-binding and tau2 transactivation domains of the rat glucocorticoid receptor constitute a nuclear matrix-targeting signal. Mol Endocrinol. (1998) 12:1420–31. 10.1210/mend.12.9.0169 9731709

[79] Black BE Holaska JM Rastinejad F Paschal BM. DNA binding domains in diverse nuclear receptors function as nuclear export signals. Curr Biol. (2001) 11:1749–58. 10.1016/S0960-9822(01)00537-1 11719216

[80] Carrigan A Walther RF Salem H Wu D Atlas E Lefebvre YA Haché: an active nuclear retention signal in the glucocorticoid receptor functions as a strong inducer of transcriptional activation. J Biol Chem. (2007) 282:10963–71. 10.1074/jbc.M602931200 17314103

[81] Lu NZ Cidlowski JA. The origin and functions of multiple human glucocorticoid receptor isoforms. Ann N Y Acad Sci. (2004) 1024:102–23. 10.1196/annals.1321.008 15265776

[82] Oakley RH Sar M Cidlowski JA. The human glucocorticoid receptor beta isoform. Expression, biochemical properties, and putative function. J Biol Chem. (1996) 271:9550–9. 10.1074/jbc.271.16.9550 8621628

[83] Oakley RH Jewell CM Yudt MR Bofetiado DM Cidlowski JA. The dominant negative activity of the human glucocorticoid receptor β isoform specificity and mechanisms of action. J Biol Chem. (1999) 274:27857–66. 10.1074/jbc.274.39.27857 10488132

[84] Charmandari E Chrousos GP Ichijo T Bhattacharyya N Vottero A Souvatzoglou E. The Human Glucocorticoid Receptor (hGR) β isoform suppresses the transcriptional activity of hGRα by interfering with formation of active coactivator complexes. Mol Endocrinol. (2005) 19:52–64. 10.1210/me.2004-0112 15459252

[85] Kino T Manoli I Kelkar S Wang Y Su YA Chrousos GP. Glucocorticoid receptor (GR) β has intrinsic, GRα-independent transcriptional activity. Biochem Biophys Res Commun. (2009) 381:671–5. 10.1016/j.bbrc.2009.02.110 19248771 PMC2796800

[86] Lewis-Tuffin LJ Jewell CM Bienstock RJ Collins JB Cidlowski JA. Human glucocorticoid receptor beta binds RU-486 and is transcriptionally active. Mol Cell Biol. (2007) 27:2266–82. 10.1128/MCB.01439-06 17242213 PMC1820503

[87] Nagy Z Acs B Butz H Feldman K Marta A Szabo PM Overexpression of GR beta in colonic mucosal cell line partly reflects altered gene expression in colonic mucosa of patients with inflammatory bowel disease. J Steroid Biochem Mol Biol. (2016) 155:76–84. 10.1016/j.jsbmb.2015.10.006 26480216

[88] Ray DW Davis JR White A Clark AJ. Glucocorticoid receptor structure and function in glucocorticoid-resistant small cell lung carcinoma cells. Cancer Res. (1996) 56:3276–80. 8764121

[89] Krett NL Pillay S Moalli PA Greipp PR Rosen ST. A variant glucocorticoid receptor messenger RNA is expressed in multiple myeloma patients. Cancer Res. (1995) 55:2727–9. 7796394

[90] Sánchez-Vega B Krett N Rosen ST Gandhi V. Glucocorticoid receptor transcriptional isoforms and resistance in multiple myeloma cells. Mol Cancer Ther. (2006) 5:3062–70. 10.1158/1535-7163.MCT-06-0344 17172408

[91] Schaaf MJ M Cidlowski JA. AUUUA motifs in the 3′UTR of human glucocorticoid receptor α and β mRNA destabilize mRNA and decrease receptor protein expression. Steroids. (2002) 67:627–36. 10.1016/S0039-128X(02)00015-6 11996936

[92] Vreugdenhil E Verissimo CS L Mariman R Kamphorst JT Barbosa JS Zweers T. MicroRNA 18 and 124a down-regulate the glucocorticoid receptor: implications for glucocorticoid responsiveness in the brain. Endocrinology. (2009) 150:2220–8. 10.1210/en.2008-1335 19131573

[93] Wang SS Mu RH Li CF Dong SQ Geng D Liu Q. microRNA-124 targets glucocorticoid receptor and is involved in depression-like behaviors. Prog Neuropsychopharmacol Biol Psychiatry. (2017) 79:417–25. 10.1016/j.pnpbp.2017.07.024 28764913

[94] Lu NZ Cidlowski JA. Translational regulatory mechanisms generate N-terminal glucocorticoid receptor isoforms with unique transcriptional target genes. Mol Cell. (2005) 18:331–42. 10.1016/j.molcel.2005.03.025 15866175

[95] Chrousos GP Kino T. Intracellular glucocorticoid signaling: a formerly simple system turns stochastic. Sci STKE. (2005) 2005:pe48. 10.1126/stke.3042005pe48 16204701

[96] Lu NZ Collins JB Grissom SF Cidlowski JA. Selective regulation of bone cell apoptosis by translational isoforms of the glucocorticoid receptor. Mol Cell Biol. (2007) 27:7143–60. 10.1128/MCB.00253-07 17682054 PMC2168898

[97] Wu I Shin SC Cao Y Bender IK Jafari N Feng G. Selective glucocorticoid receptor translational isoforms reveal glucocorticoid-induced apoptotic transcriptomes. Cell Death Dis. (2013) 4:e453. 10.1038/cddis.2012.193 23303127 PMC3563981

[98] Vandevyver S Dejager L Libert C. On the trail of the glucocorticoid receptor: into the nucleus and back. Traffic. (2011) 13:364–74. 10.1111/j.1600-0854.2011.01288.x 21951602

[99] Smith DF Toft DO. Steroid-receptors and their associated proteins. Mol Endocrinol. (1993) 7:4–11. 10.1210/mend.7.1.8446107 8446107

[100] Morishima Y Murphy PJM Li D-P Sanchez ER Pratt WB. Stepwise assembly of a glucocorticoid receptor·hsp90 heterocomplex resolves two sequential ATP-dependent events involving first hsp70 and then hsp90 in opening of the steroid binding pocket. J Biol Chem. (2000) 275:18054–60. 10.1074/jbc.M000434200 10764743

[101] Chen S Smith DF. Hop as an adaptor in the heat shock protein 70 (Hsp70) and Hsp90 chaperone machinery. J Biol Chem. (1998) 273:35194–200. 10.1074/jbc.273.52.35194 9857057

[102] Morishima Y Kanelakis KC Murphy PJM Lowe ER Jenkins GJ Osawa Y. The Hsp90 cochaperone p23 is the limiting component of the multiprotein Hsp90/Hsp70-based chaperone system *in vivo* where it acts to stabilize the client protein·Hsp90 complex. J Biol Chem. (2003) 278:48754–63. 10.1074/jbc.M309814200 14507910

[103] Riggs DL Roberts PJ Chirillo SC Cheung-Flynn J Prapapanich V Ratajczak T. The Hsp90-binding peptidylprolyl isomerase FKBP52 potentiates glucocorticoid signaling *in vivo*. EMBO J. (2003) 22:1158–67. 10.1093/emboj/cdg108 12606580 PMC150341

[104] Freedman ND Yamamoto KR. Importin 7 and importin α/importin β are nuclear import receptors for the glucocorticoid receptor. Mol Biol Cell. (2004) 15:2276–86. 10.1091/mbc.e03-11-0839 15004228 PMC404022

[105] Echeverría PC Mazaira G Erlejman A Gomez-Sanchez C Pilipuk G Galigniana MD. Nuclear import of the glucocorticoid receptor-hsp90 complex through the nuclear pore complex is mediated by its interaction with Nup62 and importin β. Mol Cell Biol. (2009) 29:4788–97. 10.1128/MCB.00649-09 19581287 PMC2725705

[106] Galigniana MD Echeverría PC Erlejman AG Piwien-Pilipuk G. Role of molecular chaperones and TPR-domain proteins in the cytoplasmic transport of steroid receptors and their passage through the nuclear pore. Nucleus. (2010) 1:299–308. 10.4161/nucl.1.4.11743 21113270 PMC2990191

[107] Holaska JM Black BE Love DC Hanover JA Leszyk J Paschal BM. Calreticulin is a receptor for nuclear export. J Cell Biol. (2001) 152:127–40. 10.1083/jcb.152.1.127 11149926 PMC2193655

[108] Holaska JM Black BE Rastinejad F Paschal BM. Ca2+-dependent nuclear export mediated by calreticulin. Mol Cell Biol. (2002) 22:6286–97. 10.1128/MCB.22.17.6286-6297.2002 12167720 PMC133999

[109] Presman DM Ganguly S Schiltz LR Johnson TA Karpova TS Hager GL. DNA binding triggers tetramerization of the glucocorticoid receptor in live cells. Proc Natl Acad Sci USA. (2016) 113:8236–41. 10.1073/pnas.1606774113 27382178 PMC4961135

[110] Presman DM Hager GL. More than meets the dimer: what is the quaternary structure of the glucocorticoid receptor? Transcription. (2016) 8:32–9. 10.1080/21541264.2016.1249045 27764575 PMC5279712

[111] Reichardt HM Kaestner KH Tuckermann J Kretz O Wessely O Bock R DNA binding of the glucocorticoid receptor is not essential for survival. Cell. (1998) 93:531–41. 10.1016/S0092-8674(00)81183-6 9604929

[112] Bledsoe RK Stewart EL Pearce KH. Structure and function of the glucocorticoid receptor ligand binding domain. Nucl Recept Coregulat. (2004) 68:49–91. 10.1016/S0083-6729(04)68002-2 15193451

[113] Bianchetti L Wassmer B Defosset A Smertina A Tiberti ML Stote RH. Alternative dimerization interfaces in the glucocorticoid receptor-alpha ligand binding domain. Biochim Biophys Acta Gen Subj. (2018) 1862:1810–25. 10.1016/j.bbagen.2018.04.022 29723544

[114] Reddy TE Pauli F Sprouse RO Neff NF Newberry KM Garabedian MJ. Genomic determination of the glucocorticoid response reveals unexpected mechanisms of gene regulation. Genome Res. (2009) 19:2163–71. 10.1101/gr.097022.109 19801529 PMC2792167

[115] John S Sabo PJ Thurman RE Sung MH Biddie SC Johnson TA. Chromatin accessibility pre-determines glucocorticoid receptor binding patterns. Nat Genet. (2011) 43:264–8. 10.1038/ng.759 21258342 PMC6386452

[116] So AY Chaivorapol C Bolton EC Li H Yamamoto KR. Determinants of cell- and gene-specific transcriptional regulation by the glucocorticoid receptor. PLoS Genet. (2007) 3:e94. 10.1371/journal.pgen.0030094 17559307 PMC1904358

[117] Schiller BJ Chodankar R Watson LC Stallcup MR Yamamoto KR. Glucocorticoid receptor binds half sites as a monomer and regulates specific target genes. Genome Biol. (2014) 15:418. 10.1186/s13059-014-0418-y 25085117 PMC4149261

[118] Diamond MI Miner JN Yoshinaga SK Yamamoto KR Transcription factor interactions: selectors of positive or negative regulation from a single DNA element. Science. (1990) 249:1266–72. 10.1126/science.2119054 2119054

[119] Lim H-W Uhlenhaut HN Rauch A Weiner J Hübner S Hübner N. Genomic redistribution of GR monomers and dimers mediates transcriptional response to exogenous glucocorticoid *in vivo*. Genome Res. (2015) 25:836–44. 10.1101/gr.188581.114 25957148 PMC4448680

[120] Hudson WH Youn C Ortlund EA. The structural basis of direct glucocorticoid-mediated transrepression. Nat Struct Mol Biol. (2012) 20:53–8. 10.1038/nsmb.2456 23222642 PMC3539207

[121] Surjit M Ganti K Mukherji A Ye T Hua G Metzger D. Widespread negative response elements mediate direct repression by agonist- liganded glucocorticoid receptor. Cell. (2011) 145:224–41. 10.1016/j.cell.2011.03.027 21496643

[122] Luecke HF Yamamoto KR. The glucocorticoid receptor blocks P-TEFb recruitment by NFκB to effect promoter-specific transcriptional repression. Genes Dev. (2005) 19:1116–27. 10.1101/gad.1297105 15879558 PMC1091745

[123] Rao NAS McCalman MT Moulos P Francoijs KJ Chatziioannou A Kolisis FN. Coactivation of GR and NFKB alters the repertoire of their binding sites and target genes. Genome Res. (2011) 21:1404–16. 10.1101/gr.118042.110 21750107 PMC3166826

[124] Weikum ER de Vera I Nwachukwu JC Hudson WH Nettles KW Kojetin DJ Tethering not required: the glucocorticoid receptor binds directly to activator protein-1 recognition motifs to repress inflammatory genes. Nucleic Acids Res. (2017) 45:8596–608. 10.1093/nar/gkx509 28591827 PMC5737878

[125] Sheppard K-A Phelps KM Williams AJ Thanos D Glass CK Rosenfeld MG. Nuclear integration of glucocorticoid receptor and nuclear factor-κB signaling by CREB-binding protein and steroid receptor coactivator-1. J Biol Chem. (1998) 273:29291–4. 10.1074/jbc.273.45.29291 9792627

[126] Bhandare R Damera G Banerjee A Flammer JR Keslacy S Rogatsky I. Glucocorticoid receptor interacting protein-1 restores glucocorticoid responsiveness in steroid-resistant airway structural cells. Am J Respir Cell Mol Biol. (2010) 42:9–15. 10.1165/rcmb.2009-0239RC 19805480 PMC2809222

[127] Kino T Chrousos GP. Tumor necrosis factor alpha receptor- and Fas-associated FLASH inhibit transcriptional activity of the glucocorticoid receptor by binding to and interfering with its interaction with p160 type nuclear receptor coactivators. J Biol Chem. (2003) 278:3023–9. 10.1074/jbc.M209234200 12477726

[128] Scheinman RI Gualberto A Jewell CM Cidlowski JA Baldwin AS. Characterization of mechanisms involved in transrepression of NF-kappa B by activated glucocorticoid receptors. Mol Cell Biol. (1995) 15:943–53. 10.1128/MCB.15.2.943 7823959 PMC231982

[129] Meijsing SH Pufall MA So AY Bates DL Chen L Yamamoto KR. DNA binding site sequence directs glucocorticoid receptor structure and activity. Science. (2009) 324:407–10. 10.1126/science.1164265 19372434 PMC2777810

[130] Thomas-Chollier M Watson LC Cooper SB Pufall MA Liu JS Borzym K. A naturally occurring insertion of a single amino acid rewires transcriptional regulation by glucocorticoid receptor isoforms. Proc Natl Acad Sci USA. (2013) 110:17826–31. 10.1073/pnas.1316235110 24127590 PMC3816441

[131] Watson LC Kuchenbecker KM Schiller BJ Gross JD Pufall MA Yamamoto KR. The glucocorticoid receptor dimer interface allosterically transmits sequence-specific DNA signals. Nat Struct Mol Biol. (2013) 20:876–83. 10.1038/nsmb.2595 23728292 PMC3702670

[132] Kauppi B Jakob C Färnegårdh M Yang J Ahola H Alarcon M. The three-dimensional structures of antagonistic and agonistic forms of the glucocorticoid receptor ligand-binding domain: RU-486 induces a transconformation that leads to active antagonism. J Biol Chem. (2003) 278:22748–54. 10.1074/jbc.M212711200 12686538

[133] Wang J-C Shah N Pantoja C Meijsing SH Ho JD Scanlan TS. Novel arylpyrazole compounds selectively modulate glucocorticoid receptor regulatory activity. Genes Dev. (2006) 20:689–99. 10.1101/gad.1400506 16543221 PMC1413289

[134] Miller AL Webb SM Copik AJ Wang Y Johnson BH Kumar R. p38 Mitogen-Activated Protein Kinase (MAPK) is a key mediator in glucocorticoid-induced apoptosis of lymphoid cells: correlation between p38 MAPK activation and site-specific phosphorylation of the human glucocorticoid receptor at serine 211. Mol Endocrinol. (2005) 19:1569–83. 10.1210/me.2004-0528 15817653

[135] Webster JC Jewell CM Bodwell JE Munck A Sar M Cidlowski JA. Mouse glucocorticoid receptor phosphorylation status influences multiple functions of the receptor protein. J Biol Chem. (1997) 272:9287–93. 10.1074/jbc.272.14.9287 9083064

[136] Krstic MD Rogatsky I Yamamoto KR Garabedian MJ. Mitogen-activated and cyclin-dependent protein kinases selectively and differentially modulate transcriptional enhancement by the glucocorticoid receptor. Mol Cell Biol. (1997) 17:3947–54. 10.1128/MCB.17.7.3947 9199329 PMC232247

[137] Wang Z Frederick J Garabedian MJ. Deciphering the phosphorylation “code” of the glucocorticoid receptor *in vivo*. J Biol Chem. (2002) 277:26573–80. 10.1074/jbc.M110530200 12000743

[138] Khan SH McLaughlin WA Kumar R. Site-specific phosphorylation regulates the structure and function of an intrinsically disordered domain of the glucocorticoid receptor. Sci Rep. (2017) 7:15440. 10.1038/s41598-017-15549-5 29133811 PMC5684351

[139] Iaili N Garabedian MJ Modulation of glucocorticoid receptor function via phosphorylation. Ann N Y Acad Sci. (2004) 1024:86–101. 10.1196/annals.1321.007 15265775

[140] Itoh M Adachi M Yasui H Takekawa M Tanaka H Imai K. Nuclear export of glucocorticoid receptor is enhanced by c-Jun N-terminal kinase-mediated phosphorylation. Mol Endocrinol. (2002) 16:2382–92. 10.1210/me.2002-0144 12351702

[141] Wallace AD Cidlowski JA. Proteasome-mediated glucocorticoid receptor degradation restricts transcriptional signaling by glucocorticoids. J Biol Chem. (2001) 276:42714–21. 10.1074/jbc.M106033200 11555652

[142] Hua G Paulen L Chambon P. GR SUMOylation and formation of an SUMO-SMRT/NCoR1-HDAC3 repressing complex is mandatory for GC-induced IR nGRE-mediated transrepression. Proc Natl Acad Sci USA. (2016) 113:E626-34. 10.1073/pnas.1522821113 26712002 PMC4747746

[143] Galigniana MD Piwien-Pilipuk G Assreuy J. Inhibition of glucocorticoid receptor binding by nitric oxide. Mol Pharmacol. (1999) 55:317–23. 10.1124/mol.55.2.317 9927624

[144] Petta I Dejager L Ballegeer M Lievens S Tavernier J Bosscher K. The interactome of the glucocorticoid receptor and its influence on the actions of glucocorticoids in combatting inflammatory and infectious diseases. Microbiol Mol Biol Rev. (2016) 80:495–522. 10.1128/MMBR.00064-15 27169854 PMC4867367

[145] Rosenfeld MG Lunyak VV Glass CK. Sensors and signals: a coactivator/corepressor/epigenetic code for integrating signal-dependent programs of transcriptional response. Genes Dev. (2006) 20:1405–28. 10.1101/gad.1424806 16751179

[146] Buttgereit F Scheffold A. Rapid glucocorticoid effects on immune cells. Steroids. (2002) 67:529–34. 10.1016/S0039-128X(01)00171-4 11960631

[147] Strehl C Gaber T Lowenberg M Hommes DW Verhaar AP Schellmann S. Origin and functional activity of the membrane-bound glucocorticoid receptor. Arthritis Rheum. (2011) 63:3779–88. 10.1002/art.30637 21898343

[148] Orchinik M Murray TF Moore FL. A corticosteroid receptor in neuronal membranes. Science. (1991) 252:1848–51. 10.1126/science.2063198 2063198

[149] Samarasinghe RA Di Maio R Volonte D Galbiati F Lewis M Romero G. Nongenomic glucocorticoid receptor action regulates gap junction intercellular communication and neural progenitor cell proliferation. Proc Natl Acad Sci USA. (2011) 108:16657–62. 10.1073/pnas.1102821108 21930911 PMC3189065

[150] Mitre-Aguilar IB Cabrera-Quintero AJ Zentella-Dehesa A. Genomic and non-genomic effects of glucocorticoids: implications for breast cancer. Int J Clin Exp Pathol. (2015) 8:1–10. 10.13140/RG.2.1.1581.0165 25755688 PMC4348864

[151] Scheller K Sekeris CE Krohne G Hock R Hansen IA Scheer U. Localization of glucocorticoid hormone receptors in mitochondria of human cells. Eur J Cell Biol. (2000) 79:299–307. 10.1078/S0171-9335(04)70033-3 10887960

[152] Moutsatsou P Psarra AMG Tsiapara A Paraskevakou H Davaris P Sekeris CE. Localization of the glucocorticoid receptor in rat brain mitochondria. Arch Biochem Biophys. (2001) 386:69–78. 10.1006/abbi.2000.2162 11361002

[153] Psarra AMG Sekeris CE. Glucocorticoids induce mitochondrial gene transcription in HepG2 cells Role of the mitochondrial glucocorticoid receptor. Biochim Biophys Acta Mol Cell Res. (2011) 1813:1814–21. 10.1016/j.bbamcr.2011.05.014 21664385

[154] Du J Wang Y Hunter R Wei YL Blumenthal R Falke C. Dynamic regulation of mitochondrial function by glucocorticoids. Proc Natl Acad Sci USA. (2009) 106:3543–8. 10.1073/pnas.0812671106 19202080 PMC2637276

[155] Du J McEwen B Manji HK. Glucocorticoid receptors modulate mitochondrial function: a novel mechanism for neuroprotection. Commun Integr Biol. (2009) 2:350–2. 10.4161/cib.2.4.8554 19721888 PMC2734045

[156] Morgan DJ Poolman TM Williamson AJ Wang Z Clark NR Ma'ayan A. Glucocorticoid receptor isoforms direct distinct mitochondrial programs to regulate ATP production. Sci Rep. (2016) 6:26419. 10.1038/srep26419 27226058 PMC4881047

[157] Sobieraj DM Baker WL. Medications for asthma. JAMA. (2018) 319:1520. 10.1001/jama.2018.3808 29634830

[158] Farmer WS Marathe KS Management of atopic dermatitis. Adv Exp Med Biol. (2017) 1027:161–77. 10.1007/978-3-319-64804-0_13 29063438

[159] Paolino S Cutolo M Pizzorni C Glucocorticoid management in rheumatoid arthritis: morning or night low dose? Reumatologia. (2017) 55:189–97. 10.5114/reum.2017.69779 29056774 PMC5647534

[160] Lattanzi S Cagnetti C Danni M Provinciali L Silvestrini M. Oral and intravenous steroids for multiple sclerosis relapse: a systematic review and meta-analysis. J Neurol. (2017) 264:1697–704. 10.1007/s00415-017-8505-0 28492970

[161] Kuhn A Bonsmann G Anders H-J Herzer P Tenbrock K Schneider M. The diagnosis and treatment of systemic lupus erythematosus. Dtsch Arztebl Int. (2015) 112:423–32. 10.3238/arztebl.2015.0423 26179016 PMC4558874

[162] Strehl C Bijlsma JWJ de Wit M Boers M Caeyers N Cutolo M. Defining conditions where long-term glucocorticoid treatment has an acceptably low level of harm to facilitate implementation of existing recommendations: viewpoints from an EULAR task force. Ann Rheum Dis. (2016) 75:952–57. 10.1136/annrheumdis-2015-208916 26933146

[163] Ozen G Pedro S Wolfe F Michaud K. Medications associated with fracture risk in patients with rheumatoid arthritis. Ann Rheum Dis. (2019). 10.1136/annrheumdis-2019-215328. [Epub ahead of print]. 31092411

[164] Nicolaides NC Charmandari E. Novel insights into the molecular mechanisms underlying generalized glucocorticoid resistance and hypersensitivity syndromes. Hormones. (2017) 16:124–38. 10.14310/horm.2002.1728 28742501

[165] Wilkinson L Verhoog NJ D Louw A Disease and treatment associated acquired glucocorticoid resistance. Endocr Connect. (2018) 7:R328–49. 10.1530/EC-18-0421 PMC6280593 30352419

[166] Ramamoorthy S Cidlowski JA. Ligand-induced repression of the glucocorticoid receptor gene is mediated by an NCoR1 repression complex formed by long-range chromatin interactions with intragenic glucocorticoid response elements. Mol Cell Biol. (2013) 33:1711–22. 10.1128/MCB.01151-12 23428870 PMC3624172

[167] Zhang BQ Zhang YD Xu TZ Yin YY Huang RR Wang YC. Chronic dexamethasone treatment results in hippocampal neurons injury due to activate NLRP1 inflammasome *in vitro*. Int Immunopharmacol. (2017) 49:222–30. 10.1016/j.intimp.2017.05.039 28605710

[168] Hodge G Roscioli E Jersmann H Tran HB Holmes M Reynolds PN. Steroid resistance in COPD is associated with impaired molecular chaperone Hsp90 expression by pro-inflammatory lymphocytes. Respir Res. (2016) 17:135. 10.1186/s12931-016-0450-4 27769261 PMC5075183

[169] Chen HB Fan JF Shou QY Zhang LZ Ma HZ Fan YS. Hypermethylation of glucocorticoid receptor gene promoter results in glucocorticoid receptor gene low expression in peripheral blood mononuclear cells of patients with systemic lupus erythematosus. Rheumatol Int. (2015) 35:1335–42. 10.1007/s00296-015-3266-5 25899090

[170] Ray A Prefontaine KE. Physical association and functional antagonism between the p65 subunit of transcription factor NF-kappa B and the glucocorticoid receptor. Proc Natl Acad Sci USA. (1994) 91:752–6. 10.1073/pnas.91.2.752 8290595 PMC43027

[171] Yang N Ray DW Matthews LC. Current concepts in glucocorticoid resistance. Steroids. (2012) 77:1041–9. 10.1016/j.steroids.2012.05.007 22728894

[172] Matthews JG Ito K Barnes PJ Adcock IM. Defective glucocorticoid receptor nuclear translocation and altered histone acetylation patterns in glucocorticoid-resistant patients. J Allergy Clin Immunol. (2004) 113:1100–8. 10.1016/j.jaci.2004.03.018 15208591

[173] Szatmáry Z Garabedian MJ Vilcek J. Inhibition of glucocorticoid receptor-mediated transcriptional activation by p38 mitogen-activated protein (MAP) kinase. J Biol Chem. (2004) 279:43708–15. 10.1074/jbc.M406568200 15292225

[174] Van Bogaert T Vandevyver S Dejager L Van Hauwermeiren F Pinheiro I Petta I. Tumor necrosis factor inhibits glucocorticoid receptor function in mice: a strong signal toward lethal shock. J Biol Chem. (2011) 286:26555–67. 10.1074/jbc.M110.212365 21646349 PMC3143620

[175] Dejager L Dendoncker K Eggermont M Souffriau J Van Hauwermeiren F Willart M. Neutralizing TNFalpha restores glucocorticoid sensitivity in a mouse model of neutrophilic airway inflammation. Mucosal Immunol. (2015) 8:1212–25. 10.1038/mi.2015.12 25760421

[176] Duma D Silva-Santos J Assreuy J. Inhibition of glucocorticoid receptor binding by nitric oxide in endotoxemic rats^*^. Crit Care Med. (2004) 32:2304. 10.1097/01.CCM.0000145996.57901.D7 15640646

[177] Barnes PJ Adcock IM. Glucocorticoid resistance in inflammatory diseases. Lancet. (2009) 373:1905–17. 10.1016/S0140-6736(09)60326-3 19482216

[178] Rodriguez JM Monsalves-Alvarez M Henriquez S Llanos MN Troncoso R. Glucocorticoid resistance in chronic diseases. Steroids. (2016) 115:182–92. 10.1016/j.steroids.2016.09.010 27643454

[179] Diver S Russell RJ Brightling CE. New and emerging drug treatments for severe asthma. Clin Exp Allergy. (2018) 48:241–52. 10.1111/cea.13086 29315966

[180] Smolen JS Landewé R Bijlsma J Burmester G Chatzidionysiou K Dougados M. EULAR recommendations for the management of rheumatoid arthritis with synthetic and biological disease-modifying antirheumatic drugs: 2016 update. Ann Rheum Dis. (2017) 76:960–77. 10.1136/annrheumdis-2016-210715 28264816

[181] Palmowski Y Buttgereit T Dejaco C Bijlsma JW Matteson EL Voshaar M. “Official View” on glucocorticoids in rheumatoid arthritis: a systematic review of international guidelines and consensus statements. Arthritis Care Res. (2017) 69:1134–41. 10.1002/acr.23185 28029750

[182] Duru N van der Goes MC Jacobs JWG Andrews T Boers M Buttgereit F. EULAR evidence-based and consensus-based recommendations on the management of medium to high-dose glucocorticoid therapy in rheumatic diseases. Ann Rheum Dis. (2013) 72:1905–13. 10.1136/annrheumdis-2013-203249 23873876

[183] van der Goes MC Jacobs JWG Boers M Andrews T Blom-Bakkers MAM Buttgereit F. Monitoring adverse events of low-dose glucocorticoid therapy: EULAR recommendations for clinical trials and daily practice. Ann Rheum Dis. (2010) 69:1913–9. 10.1136/ard.2009.124958 20693273

[184] Buttgereit F Spies CM Bijlsma JWJ. Novel glucocorticoids: where are we now and where do we want to go? Clin Exp Rheumatol. (2015) 33(4 Suppl 92):33. 26457359

[185] Buttgereit F Bijlsma J Strehl C. Will we ever have better glucocorticoids? Clin Immunol. (2018) 186:64–6. 10.1016/j.clim.2017.07.023 28757452

[186] Vandewalle J Luypaert A Bosscher K Libert C. Therapeutic mechanisms of glucocorticoids. Trends Endocrinol Metab. (2018) 29:42–54. 10.1016/j.tem.2017.10.010 29162310

[187] Reichardt HM Tuckermann JP Göttlicher M Vujic M Weih F Angel P. Repression of inflammatory responses in the absence of DNA binding by the glucocorticoid receptor. EMBO J. (2001) 20:7168–73. 10.1093/emboj/20.24.7168 11742993 PMC125338

[188] Presman DM Ogara MF Stortz M Alvarez LD Pooley JR Schiltz RL. Live cell imaging unveils multiple domain requirements for *in vivo* dimerization of the glucocorticoid receptor. PLoS Biol. (2014) 12:e1001813. 10.1371/journal.pbio.1001813 24642507 PMC3958349

[189] Heck S Kullmann M Gast A Ponta H Rahmsdorf HJ Herrlich P. A distinct modulating domain in glucocorticoid receptor monomers in the repression of activity of the transcription factor AP-1. EMBO J. (1994) 13:4087–95. 10.1002/j.1460-2075.1994.tb06726.x 8076604 PMC395330

[190] Adams M Meijer OC Wang JA Bhargava A Pearce D Homodimerization of the glucocorticoid receptor is not essential for response element binding: activation of the phenylethanolamine N-methyltransferase gene by dimerization-defective mutants. Mol Endocrinol. (2003) 17:2583–92. 10.1210/me.2002-0305 12933902

[191] Jewell CM Scoltock AB Hamel BL Yudt MR Cidlowski JA. Complex human glucocorticoid receptor dim mutations define glucocorticoid induced apoptotic resistance in bone cells. Mol Endocrinol. (2012) 26:244–56. 10.1210/me.2011-1116 22174376 PMC3275167

[192] Ballegeer M Looveren K Timmermans S Eggermont M Vandevyver S Thery F. Glucocorticoid receptor dimers control intestinal STAT1 and TNF-induced inflammation in mice. J Clin Investig. (2018) 128:3265–79. 10.1172/JCI96636 29746256 PMC6063488

[193] Schäcke H Berger M Hansson TG McKerrecher D Rehwinkel H Dissociated non-steroidal glucocorticoid receptor modulators: an update on new compounds. Expert Opin Ther Patents. (2008) 18:339–52. 10.1517/13543776.18.3.339

[194] Schäcke H Berger M Rehwinkel H Asadullah K. Selective glucocorticoid receptor agonists (SEGRAs): novel ligands with an improved therapeutic index. Mol Cell Endocrinol. (2007) 275:109–17. 10.1016/j.mce.2007.05.014 17630119

[195] De Bosscher K. Selective glucocorticoid receptor modulators. J Steroid Biochem Mol Biol. (2010) 120:96–104. 10.1016/j.jsbmb.2010.02.027 20206690

[196] De Bosscher K Haegeman G Elewaut D. Targeting inflammation using selective glucocorticoid receptor modulators. Curr Opin Pharmacol. (2010) 10:497–504. 10.1016/j.coph.2010.04.007 20493772

[197] Sundahl N Bridelance J Libert C De Bosscher K Beck IM. Selective glucocorticoid receptor modulation: new directions with non-steroidal scaffolds. Pharmacol Ther. (2015) 152:28–41. 10.1016/j.pharmthera.2015.05.001 25958032

[198] De Bosscher K Vanden Berghe W Beck IM Van Molle W Hennuyer N Hapgood J. A fully dissociated compound of plant origin for inflammatory gene repression. Proc Natl Acad Sci USA. (2005) 102:15827–32. 10.1073/pnas.0505554102 16243974 PMC1276063

[199] Dewint P Gossye V De Bosscher K Vanden Berghe W Van Beneden K Deforce D. A plant-derived ligand favoring monomeric glucocorticoid receptor conformation with impaired transactivation potential attenuates collagen-induced arthritis. J Immunol. (2008) 180:2608–15. 10.4049/jimmunol.180.4.2608 18250472

[200] Schäcke H Zollner TM Döcke WD Rehwinkel H Jaroch S Skuballa W. Characterization of ZK 245186, a novel, selective glucocorticoid receptor agonist for the topical treatment of inflammatory skin diseases. Br J Pharmacol. (2009) 158:1088–103. 10.1111/j.1476-5381.2009.00238.x 19422381 PMC2785530

[201] Schäcke H Schottelius A Döcke WD Strehlke P Jaroch S Schmees N. Dissociation of transactivation from transrepression by a selective glucocorticoid receptor agonist leads to separation of therapeutic effects from side effects. Proc Natl Acad Sci USA. (2003) 101:227–32. 10.1073/pnas.0300372101 14694204 PMC314167

[202] López FJ Ardecky RJ Bebo B Benbatoul K De Grandpre L Liu S. LGD-5552, an antiinflammatory glucocorticoid receptor ligand with reduced side effects, *in vivo*. Endocrinology. (2008) 149:2080–9. 10.1210/en.2007-1353 18218700

[203] Miner JN Ardecky B Benbatoul K Griffiths K Larson CJ Mais DE. Antiinflammatory glucocorticoid receptor ligand with reduced side effects exhibits an altered protein-protein interaction profile. Proc Natl Acad Sci USA. (2007) 104:19244–9. 10.1073/pnas.0705517104 18032610 PMC2148275

[204] Owen HC Miner JN Ahmed SF Farquharson C. The growth plate sparing effects of the selective glucocorticoid receptor modulator, AL-438. Mol Cell Endocrinol. (2006) 264:164–70. 10.1016/j.mce.2006.11.006 17182172

[205] Coghlan MJ Jacobson PB Lane B Nakane M Lin C Elmore SW. A novel antiinflammatory maintains glucocorticoid efficacy with reduced side effects. Mol Endocrinol. (2003) 17:860–9. 10.1210/me.2002-0355 12586843

[206] van Loo G Sze M Bougarne N Praet J Guire C Ullrich A. Antiinflammatory properties of a plant-derived nonsteroidal, dissociated glucocorticoid receptor modulator in experimental autoimmune encephalomyelitis. Mol Endocrinol. (2009) 24:310–22. 10.1210/me.2009-0236 19965930 PMC5428123

[207] Miyoshi S Hey-Hadavi J Nagaoka M Tammara B. Pharmacokinetics and food-effect of fosdagrocorat (PF-04171327), a dissociated agonist of the glucocorticoid receptor, in healthy adult Caucasian and Japanese subjects. Int J Clin Pharmacol Ther. (2016) 54:966–76. 10.5414/CP202659 27781421

[208] Stock T Fleishaker D Wang X Mukherjee A Mebus C. Improved disease activity with fosdagrocorat (PF-04171327), a partial agonist of the glucocorticoid receptor, in patients with rheumatoid arthritis: a Phase 2 randomized study. Int J Rheum Dis. (2017) 20:960–70. 10.1111/1756-185X.13053 28328159 PMC6084298

[209] Weatherley B McFadyen L Tammara B. Population pharmacokinetics of fosdagrocorat (PF-04171327), a dissociated glucocorticoid receptor agonist, in patients with rheumatoid arthritis. Clin Transl Sci. (2018) 11:54–62. 10.1111/cts.12515 29106053 PMC5759734

[210] Thomsen K Møller H Graversen J Magnusson NE Moestrup SK Vilstrup H. Anti-CD163-dexamethasone conjugate inhibits the acute phase response to lipopolysaccharide in rats. World J Hepatol. (2016) 8:726–30. 10.4254/wjh.v8.i17.726 27330681 PMC4911506

[211] Metselaar JM Wauben MHM Wagenaar-Hilbers JPA Boerman OC Storm G. Complete remission of experimental arthritis by joint targeting of glucocorticoids with long-circulating liposomes. Arthritis Rheum. (2003) 48:2059–66. 10.1002/art.11140 12847701

[212] Pinheiro I Dejager L Petta I Vandevyver S Puimege L Mahieu T. LPS resistance of SPRET/Ei mice is mediated by Gilz, encoded by the Tsc22d3 gene on the X chromosome. EMBO Mol Med. (2013) 5:456–70. 10.1002/emmm.201201683 23495141 PMC3598084

[213] Bougarne N Paumelle R Caron S Hennuyer N Mansouri R Gervois P. PPARα blocks glucocorticoid receptor α-mediated transactivation but cooperates with the activated glucocorticoid receptor α for transrepression on NF-κB. Proc Natl Acad Sci. (2009) 106:7397–402. 10.1073/pnas.0806742106 19376972 PMC2678648

[214] Deckers J Bougarne N Mylka V Desmet S Luypaert A Devos M. Co-activation of glucocorticoid receptor and peroxisome proliferator–activated receptor-γ in murine skin prevents worsening of atopic march. J Investig Dermatol. (2018) 138:1360–70. 10.1016/j.jid.2017.12.023 29288652 PMC7611015

[215] Rauch A Seitz S Baschant U Schilling AF Illing A Stride B. Glucocorticoids suppress bone formation by attenuating osteoblast differentiation via the monomeric glucocorticoid receptor. Cell Metab. (2010) 11:517–31. 10.1016/j.cmet.2010.05.005 20519123

[216] Waddell DS Baehr LM van den Brandt J Johnsen SA Reichardt HM Furlow JD. The glucocorticoid receptor and FOXO1 synergistically activate the skeletal muscle atrophy-associated MuRF1 gene. Am J Physiol Endocrinol Metab. (2008) 295:E785–97. 10.1152/ajpendo.00646.2007 18612045 PMC2652500

[217] Vandevyver S Dejager L Van Bogaert T Kleyman A Liu Y Tuckermann J Glucocorticoid receptor dimerization induces MKP1 to protect against TNF-induced inflammation. Cytokine. (2012) 59:518 10.1016/j.cyto.2012.06.082 PMC3366401 22585571

[218] Kleiman A Hübner S Rodriguez Parkitna JM Neumann A Hofer S Weigand MA. Glucocorticoid receptor dimerization is required for survival in septic shock via suppression of interleukin-1 in macrophages. FASEB J. (2011) 26:722–9. 10.1096/fj.11-192112 22042221

[219] De Bosscher K Beck IM Dejager L Bougarne N Gaigneaux A Chateauvieux S. Selective modulation of the glucocorticoid receptor can distinguish between transrepression of NF-κB and AP-1. Cell Mol Life Sci. (2013) 71:143–63. 10.1007/s00018-013-1367-4 23784308 PMC3889831

[220] Wilkinson L Verhoog N Louw A. Novel role for receptor dimerization in post-translational processing and turnover of the GRα. Sci Rep. (2018) 8:14266. 10.1038/s41598-018-32440-z 30250038 PMC6155283

[221] De Bosscher K Beck IM Ratman D Berghe WV Libert C. Activation of the glucocorticoid receptor in acute inflammation: the SEDIGRAM concept. Trends Pharmacol Sci. (2015) 37:4–16. 10.1016/j.tips.2015.09.002 26603477

[222] Frijters R Fleuren W Toonen EJM Tuckermann JP Reichardt HM van der Maaden H Prednisolone-induced differential gene expression in mouse liver carrying wild type or a dimerization-defective glucocorticoid receptor. BMC Genomics. (2010) 11:359 10.1186/1471-2164-11-359 20525385 PMC2895630

